# Genetic diversity and phylogenetic characteristics of Chinese Tibetan and Yi minority ethnic groups revealed by non-CODIS STR markers

**DOI:** 10.1038/s41598-018-24291-5

**Published:** 2018-04-12

**Authors:** Guanglin He, Zheng Wang, Xing Zou, Xu Chen, Jing Liu, Mengge Wang, Yiping Hou

**Affiliations:** 1 Institute of Forensic Medicine, West China School of Basic Medical Sciences & Forensic Medicine, Sichuan University, Chengdu, Sichuan China; 2 Department of Forensic Medicine, College of Basic Medicine, Chongqing Medical University, Chongqing, China; 3 Department of Clinical Laboratory, the First People’s Hospital of Liangshan Yi Autonomous Prefecture, Xichang, Sichuan China

## Abstract

Non-CODIS STRs, with high polymorphism and allele frequency difference among ethnically and geographically different populations, play a crucial role in population genetics, molecular anthropology, and human forensics. In this work, 332 unrelated individuals from Sichuan Province (237 Tibetan individuals and 95 Yi individuals) are firstly genotyped with 21 non-CODIS autosomal STRs, and phylogenetic relationships with 26 previously investigated populations (9,444 individuals) are subsequently explored. In the Sichuan Tibetan and Yi, the combined power of discrimination (CPD) values are 0.9999999999999999999 and 0.9999999999999999993, and the combined power of exclusion (CPE) values are 0. 999997 and 0.999999, respectively. Analysis of molecular variance (AMOVA), principal component analysis (PCA), multidimensional scaling plots (MDS) and phylogenetic analysis demonstrated that Sichuan Tibetan has a close genetic relationship with Tibet Tibetan, and Sichuan Yi has a genetic affinity with Yunnan Bai group. Furthermore, significant genetic differences have widely existed between Chinese minorities (most prominently for Tibetan and Kazakh) and Han groups, but no population stratifications rather a homogenous group among Han populations distributed in Northern and Southern China are observed. Aforementioned results suggested that these 21 STRs are highly polymorphic and informative in the Sichuan Tibetan and Yi, which are suitable for population genetics and forensic applications.

## Introduction

Short tandem repeats (STRs) are DNA sequences with a number of tandemly repeated short sequence motifs (2–6 bp), such as (ATCT)_n_^1–3^. In the past decades, comprehensive recognition of human STRs has provided insights into anthropology, archaeology, human forensics and population genetics^4,5^. Recently, non-Combined DNA Index System short tandem repeats (non-CODIS STRs) are attractive to genetic applications like population stratification analysis, regional population structure studies and forensic individual identification and paternity testing^6–12^. Non-CODIS STRs combined with previous commercial CODIS STRs amplification systems play an indispensable complementary role in the forensic applications: disentangling missing person cases, identifying victims, and solving the parentage testing cases with mutation. Genetic diversity of non-CODIS STRs in major ethnic groups in the East Asia, especially in China, has been explored^7,8,13–17^. However, the genetic architecture of geographically and linguistically distinct populations in Sichuan Province, the 5th largest and 3rd most populous province in China, remains uncharacterized.

Sichuan consists of two geographically distinct parts: the eastern part is mostly within the fertile Sichuan Basin and the western part consists of the numerous mountain range. Han Chinese (the majority of the province’s population) mainly reside in the eastern portion, while significant minorities of Yi, Tibetan and Qiang people reside in the western portion that is impacted by inclement weather and natural disasters. Yi population, also known as Lolo population, is the seventh largest of the Chinese officially recognized 55 ethnic minority groups and the Yi is the largest ethnic minority group in Sichuan^18,19^. The population history of the Yi group remains controversial^18–20^. The main point is that the Yi migrated from southeastern Tibet through Sichuan and into the Yunnan Province and has the common ancestor with the Tibetan, Nakhi and Qiang peoples. Tibetan population is mainly resided throughout the Qinghai-Tibetan Plateau for hundreds of generations has genetic adaptations of distinct combinations of phenotype in high-altitude (>4000 m)^21–23^. Chengdu, the capital of Sichuan Province, is home to a large community of Tibetans, with 30,000 permanent Tibetan residents and up to 200,000 Tibetan floating population. In our previous study^24^, we have investigated the genetic polymorphism data of non-CODIS STRs in Sichuan Han population. However, until now no genetic diversity data about non-CODIS STRs was available for the Tibetan population and Yi population from Sichuan Province.

In continuation to our previous study^24^, the present study characterizes the genetic diversity of 21 non-CODIS STRs in Tibetan population (237 individuals) and Yi population (95 individuals) from Sichuan Province. Furthermore, other genetic data of 9,444 previously investigated individuals^6–17,24–37^ from 26 populations is used to investigate Sichuan and Chinese population genetic substructure using analysis of molecular variance (AMOVA), principal component analysis (PCA), multidimensional scaling plots (MDS) and phylogenetic analysis.

## Results

### Genetic parameters of the 21 non-CODIS STRs

Population genetic structures in East Asia are complex, especially in Chinese population stratification consisted of 56 Chinese officially recognized ethnic groups widely distributed in 34 administrative divisions. Different ethnic origin has been believed to have their special ethnic origin or common ancestors. Clearly understanding the genetic variation and forensic characteristics of different ethnic populations is indispensable in the forensic applications, especially in the paternity testing and individual identification. In the present study, a total of 332 unrelated individuals residing in Sichuan Province are genotyped using a multiplex assay amplifying 21 non-CODIS autosomal STR loci (AGCU 21 + 1 System). The detailed genotypes of 237 Tibetan individuals and 95 Yi individuals are presented respectively in Supplementary Tables S1 and S2. Forensic parameters including observed heterozygosity (Ho), expected heterozygosity (He), polymorphism information content (PIC), power of discrimination (PD), power of exclusion (PE) and typical paternity index (TPI) for each locus in two ethnic groups are shown in Table 1. No significant deviations from Hardy–Weinberg equilibrium (HWE) are observed for any of the 21 non-CODIS STRs or in two ethnic groups after Bonferroni correction (p > 0.0024). No significant deviations from linkage disequilibrium between pairwise STR loci (378 pairwise groups) are observed after Bonferroni correction (p > 0.0002) with the exception of pairwise groups between D11S4463 and D5S2500 in Tibetan population, as well as D10S1248 and D6S1017 in Yi population (Supplementary Tables S3 and S4).

**Table 1:** The forensic statistical parameters of 21 non-CODIS STR loci included in AGCU X19 PCR amplification kit in Sichuan Tibetan and Yi populations.

Populations	Tibetan							Yi						
Parameters	TPI	PD	PIC	PE	Ho	He	p	TPI	PD	PIC	PE	Ho	He	p
D6S474	1.4277	0.8488	0.6296	0.3549	0.6498	0.6864	0.2243	1.9000	0.8472	0.6436	0.4875	0.7368	0.6965	0.3920
D12ATA63	1.8810	0.8746	0.6704	0.4831	0.7342	0.7147	0.5060	2.6389	0.8700	0.6978	0.6187	0.8105	0.7458	0.1474
D22S1045	1.8516	0.8904	0.7090	0.4761	0.7300	0.7533	0.4050	2.2619	0.8742	0.7037	0.5606	0.7790	0.7520	0.5429
D10S1248	2.1161	0.8984	0.7217	0.5335	0.7637	0.7599	0.8900	2.0652	0.9268	0.7982	0.5234	0.7579	0.8260	0.0802
D1S1677	1.3022	0.8208	0.5909	0.3106	0.6160	0.6457	0.3393	1.1875	0.8086	0.5699	0.2664	0.5790	0.6339	0.2665
D11S4463	2.5213	0.9195	0.7570	0.6022	0.8017	0.7906	0.6760	1.8269	0.9086	0.7315	0.4701	0.7263	0.7727	0.2806
D1S1627	1.2474	0.7318	0.5084	0.2899	0.5992	0.5905	0.7854	1.4844	0.8472	0.6341	0.3736	0.6632	0.6805	0.7173
D3S4529	1.7955	0.8866	0.6929	0.4623	0.7215	0.7401	0.5134	2.1591	0.8889	0.7151	0.5418	0.7684	0.7616	0.8755
D2S441	2.1944	0.8999	0.7176	0.5484	0.7722	0.7573	0.5925	1.9792	0.9090	0.7492	0.5053	0.7474	0.7850	0.3725
D6S1017	1.8231	0.8984	0.7121	0.4692	0.7257	0.7548	0.2988	2.1591	0.8665	0.6783	0.5418	0.7684	0.7301	0.4002
D4S2408	1.8231	0.8828	0.6849	0.4692	0.7257	0.7333	0.7913	1.8269	0.8691	0.6716	0.4701	0.7263	0.7244	0.9671
D19S433	2.6333	0.9452	0.8039	0.6180	0.8101	0.8275	0.4798	1.8269	0.9394	0.7838	0.4701	0.7263	0.8116	0.0336
D17S1301	1.3466	0.8283	0.6013	0.3267	0.6287	0.6436	0.6327	2.0652	0.8758	0.7007	0.5234	0.7579	0.7414	0.7127
D1GATA113	1.3941	0.8186	0.5895	0.3435	0.6414	0.6561	0.6323	1.6964	0.8031	0.5946	0.4364	0.7053	0.6598	0.3492
D18S853	1.6233	0.8388	0.6226	0.4160	0.6920	0.6690	0.4518	2.1591	0.8869	0.6971	0.5418	0.7684	0.7367	0.4823
D20S482	1.6014	0.8598	0.6571	0.4096	0.6878	0.7003	0.6741	1.6964	0.8660	0.6748	0.4364	0.7053	0.7175	0.7917
D14S1434	1.4277	0.8414	0.6179	0.3549	0.6498	0.6609	0.7180	1.3971	0.8366	0.6172	0.3445	0.6421	0.6672	0.6042
D9S1122	1.6458	0.8440	0.6412	0.4224	0.6962	0.6913	0.8704	1.8269	0.8421	0.6425	0.4701	0.7263	0.6967	0.5298
D2S1776	1.9750	0.9204	0.7447	0.5044	0.7468	0.7779	0.2495	1.4844	0.9146	0.7433	0.3736	0.6632	0.7797	0.0061
D10S1435	1.9113	0.8996	0.7137	0.4901	0.7384	0.7544	0.5680	2.3750	0.8767	0.7045	0.5797	0.7895	0.7509	0.3844
D5S2500	1.6690	0.8749	0.6722	0.4289	0.7004	0.7229	0.4386	1.6379	0.8709	0.6676	0.4202	0.6947	0.7225	0.5452

TPI: Typical Paternity Index, PD: Power of Discrimination, PIC: Polymorphism Information Content, PE, Power of Exclusion, Ho: observed Heterozygosity, He, expected Heterozygosity, p: the probability of the Hardy-Weinberg testing.

In Sichuan Tibetan population, a total of 183 alleles are identified with corresponding frequencies vary from 0.0021 to 0.5401 (Supplementary Table S5). D19S433 is detected with the 15 alleles at the maximum, while D1S1627 is only detected with 6 alleles (Supplementary Figure S1). The TPI spans from 1.2474 at locus of D1S1627 to 2.6333 at locus of D19S433. The observed heterozygosity ranges from 0.5992 (D1S1627) to 0. 8101 (D19S433) with an average of 0.7258. The first three loci with highest PD are D19S433, D2S1776, and D11S4463, and the combined power of discrimination (CPD) value is 0.9999999999999999999. The highest and lowest PE loci are D19S433 (0.6180) and D1S1627 (0.2899), respectively, and the combined power of exclusion (CPE) value is 0. 999997.

In Sichuan Yi population, a total of 149 alleles are observed with corresponding allele frequencies span from 0.0053 to 0.5053 (Supplementary Table S6). As shown in Supplementary Figure S2, only 5 alleles are observed at four loci (D3S4529, D6S1017, D4S2408, and D4S2408), while D10S1248 and D19S433 are detected the most variations with 10 alleles observed. The TPI spans from 1.1875 at locus of D1S1677 to 2.6389 at locus of D12ATA63. The observed heterozygosity ranges from 0.5790 (D1S1677) to 0. 8105 (D12ATA63) with an average of 0.7258. The CPD and CPE in Yi population are 0.9999999999999999993 and 0.999999, respectively.

### Population pairwise differences

To explore the genetic similarities and differences between the two investigated populations (Sichuan Tibetan and Sichuan Yi) and 26 previously studied populations, we first calculated the F_st_ and corresponding p values via analysis of molecular variance. For Sichuan Tibetan (Supplementary Table S7), Hainan Li shows significant genetic differences at five loci, followed by Guangdong Han, Hunan Han, Zhejiang Han at three loci; Aksu Uyghur, Fujian She, Inner Mongolia Mongolian, Northern Han, Yunnan Yi, Inner Mongolia Russian at two loci; Qinghai Salar, Hubei Tujia, Ningxia Han, Guanzhong Han, Huadong Han, Beijing Han, Shandong Han, Liaoning Han, two Henan Han groups, Xinjiang Xibe, Sichuan Han and Sichuan Yi at one locus. No significant genetic differences between Sichuan Tibetan and Lhasa Tibetan, Yunnan Bai and Ili Kazakh are observed. The F_st_ and corresponding p values between Sichuan Yi and other reference populations are listed in Supplementary Table S8. Fujian She and Zhejiang Han show genetic differences with Sichuan Yi at two loci, followed by Qinghai Salar, Inner Mongolia Mongolian, Northern Han, Guanzhong Han, Yunnan Yi, Guangdong Han, Hunan Han, Beijing Han, Inner Mongolia Russian, Shandong Han, Henan Han1, Xinjiang Xibe at one locus. No difference is identified with other populations.

### Principal component analysis

**Figure 1:**
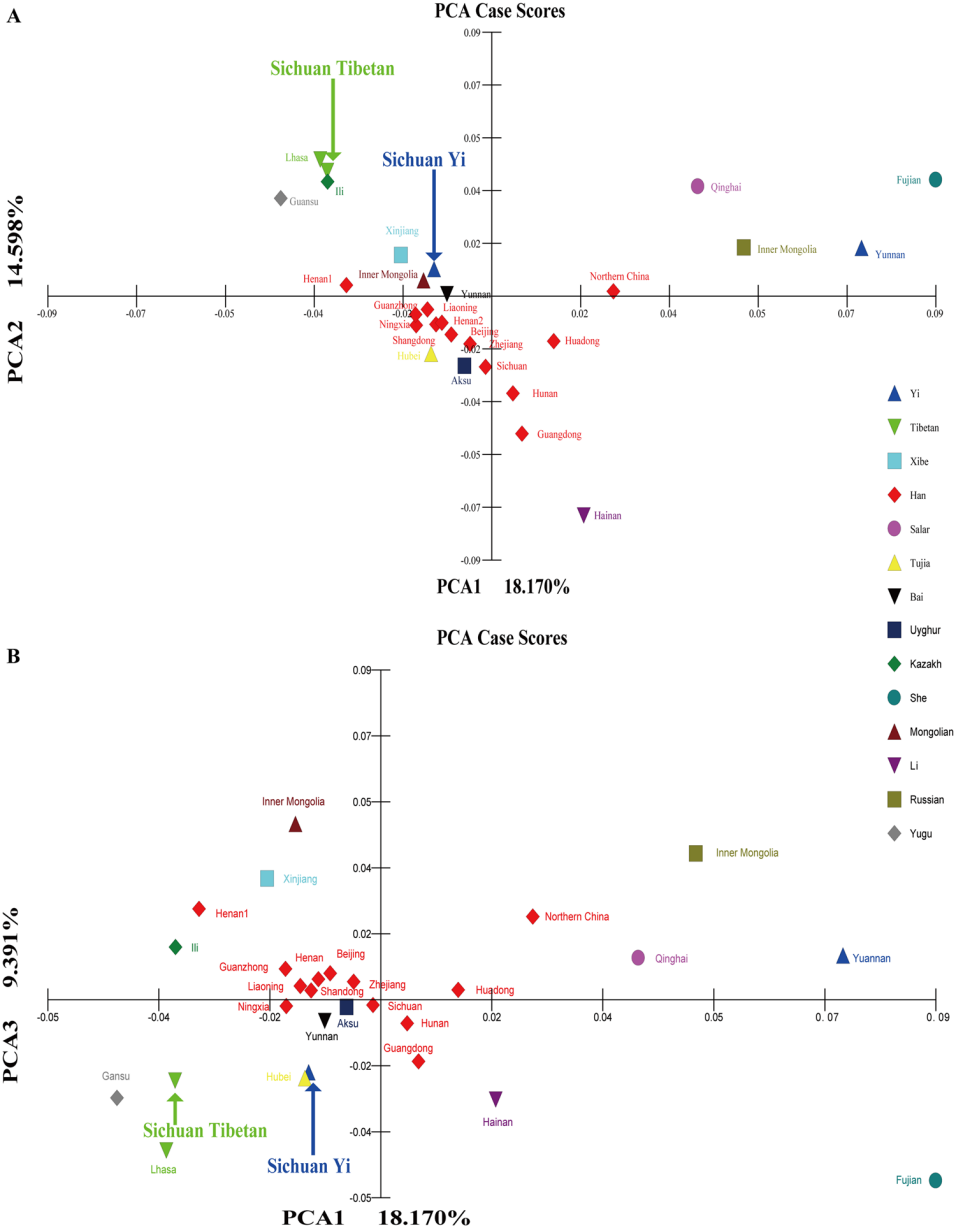
The principal component analysis (PCA) illustrated Chinese population genetic structure among 28 Chinese populations. (**A**) PCA is built based on the variance of the first and second components; (**B**) PCA constructed on the basis of the first and the third components. Each population is represented by one graphic symbol and the color label corresponding to ethnicity origin.

PCA dissected the major factors accounting for the total variances of 28 Chinese populations using the Multivariate Statistical Package version 3.22 (MVSP) and SPSS software. Figure 1 presents the PCA results for the investigated Sichuan Tibetan, Yi, and 26 Chinese reference populations, and the first three components account for 42.159% of total variances. The PCA1 is made up of 18.17% and can clearly separate Qinghai Salar, Inner Mongolia Russian, Yunnan Yi and Fujian She from other 24 reference populations. The PCA2 accounts for 14.598% and shows a clear distinction between six populations (Hainan Li, Lhasa Tibetan, Sichuan Tibetan, Fujian She, Ili Kazakh and Qinghai Salar) and others, as shown in Fig. 1A and Supplementary Figure S3. In the PCA3 (being made up of 9.391%), the cluster shows a separation between Fujian She, Lhasa Tibetan, Hainan Li, Gansu Yugu, Sichuan Tibetan, as well as Inner Mongolia Mongolian and Russian, Xinjiang Xibe and other Chinese populations (Fig. 1B and Supplementary Figure S3). Next, PCA based on the allele frequency variations of 21 non-CODIS STRs using the different calculated formula implemented in SPSS is listed in Supplementary Figure S4, which displays the first two principal components which account for 97.299% of the total variations. The population distribution pattern is consistent with the results revealed using the MVSP software and shows a clear separation between minorities and Han Chinese populations.

### Multidimensional scaling analysis

**Figure 2:**
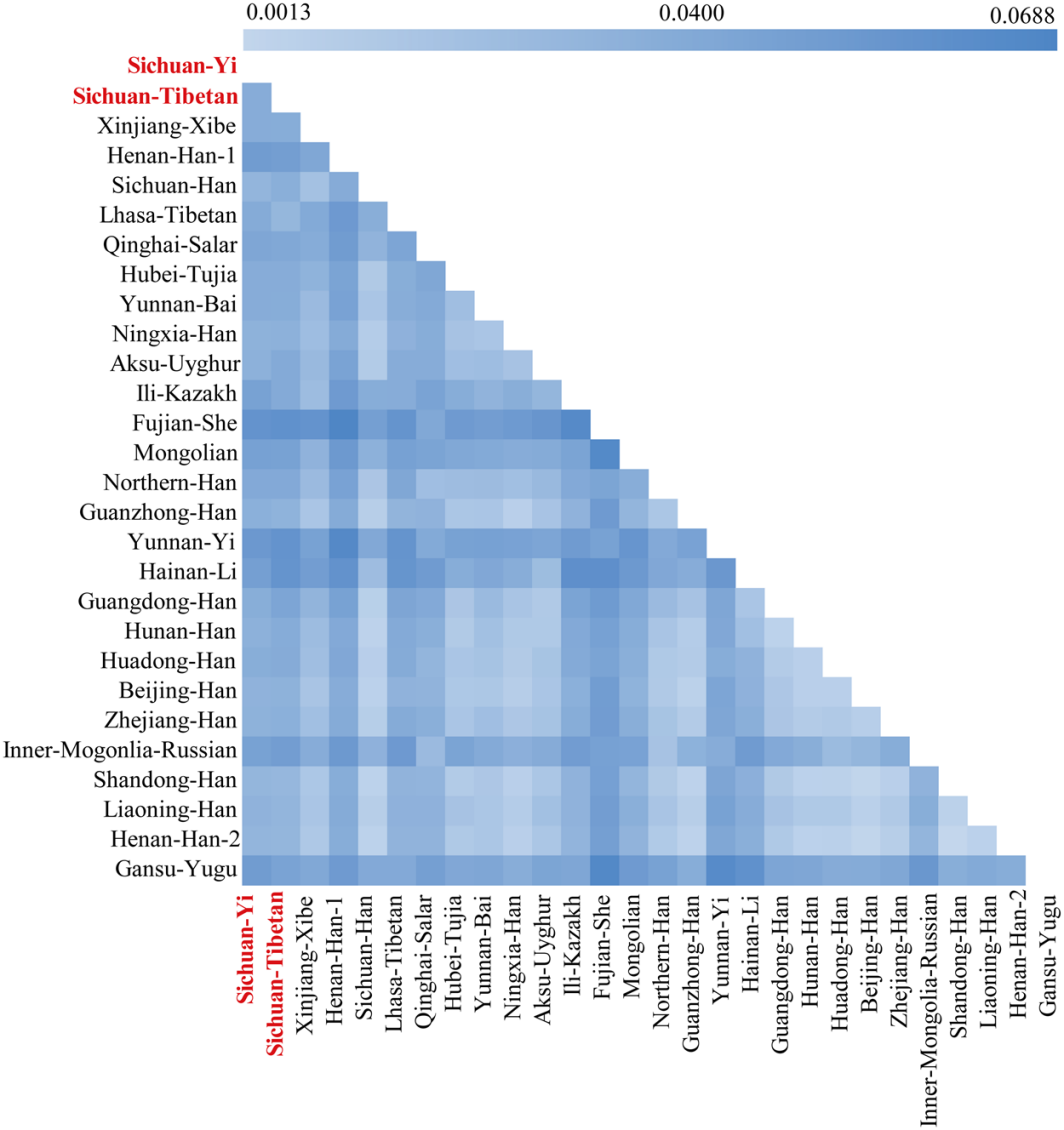
The plots of Nei’s genetic distance of Sichuan Tibetan, Sichuan Yi and 26 Chinese reference populations.

Subsequently, to evaluate the proportion of genetic heterogeneity and homogeneity attributable to Chinese population stratification, we calculated the Nei’s genetic distances for total 378 pairwise groups (Supplementary Table S9 and Fig. 2). The largest Nei’s standard genetic distance is observed between the Henan Han1 and Fujian She, and the relatively small genetic distances are identified between the Han populations distributed in different administrative divisions (0.0013 for Shandong Han and Henan2, 0.0018 for Beijing Han vs. Shandong Han, and Beijing Han vs. Henan Han2). For our studied two populations, Sichuan Yi has a relative far genetic relationship with Fujian She (0.0552) and a close genetically relationship with Shandong Han (0.0216) with a mean of 0.0316 ± 0.0095. In addition, Sichuan Tibetan has a far relationship with Fujian She (0.0577) and a close relationship with Lhasa Tibetan (0.0209) and Shandong Han (0.0208) with a mean of 0.0322 ± 0.0105.

**Figure 3:**
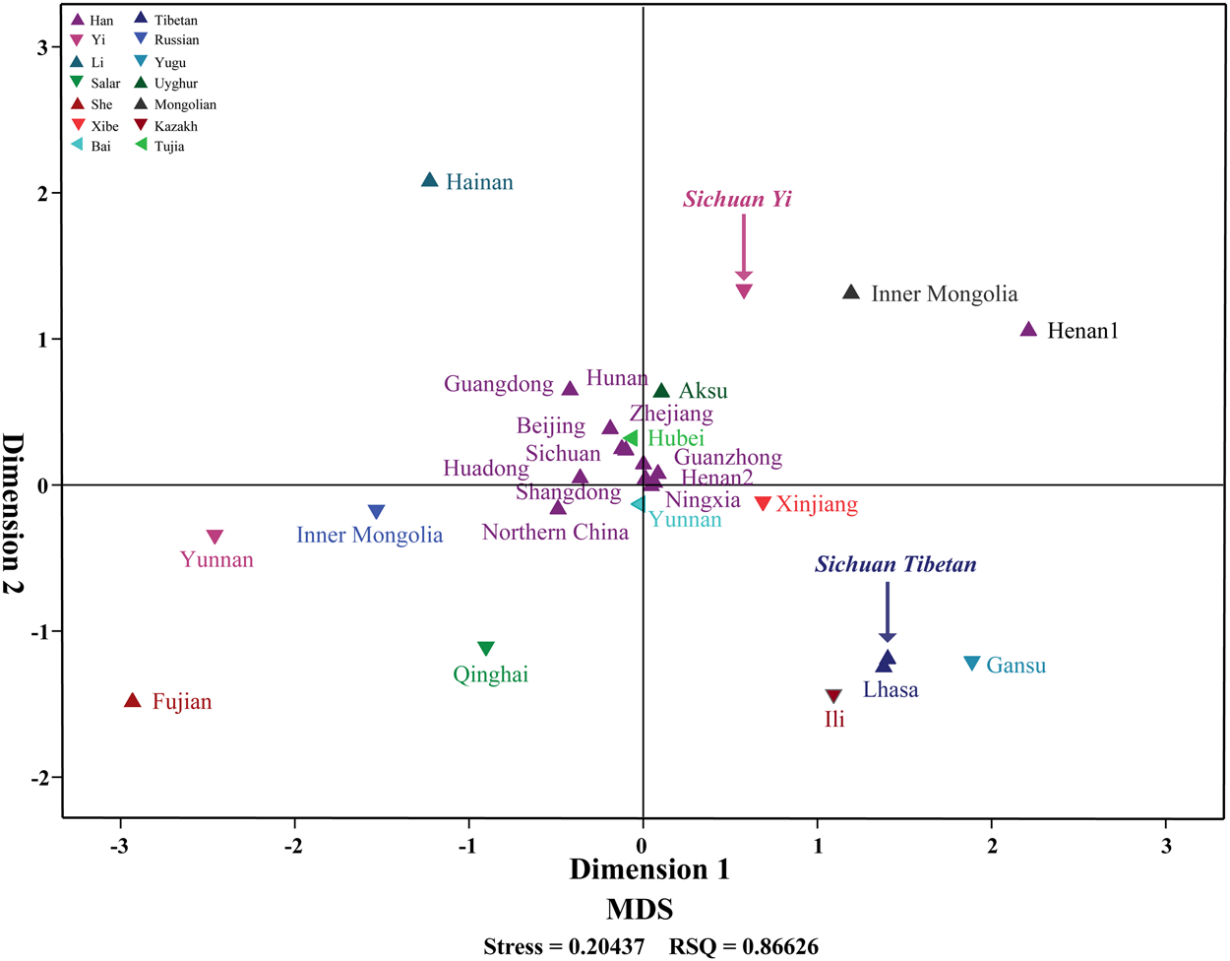
The multidimensional scaling plot (MDS) showed the genetic relationships between the Sichuan Tibetan and Yi populations and neighboring Chinese populations. MDS plots have been established on the basis of the Nei’s genetic distance. Each population is represented by one triangle and the color label corresponding to ethnicity. The population labels are in line with the Supplementary Table S9.

We next constructed a multidimensional scaling plots based on Nei’s genetic matrix to explore the population genetic structure in our 9,776 individuals. As shown in Fig. 3, all Han Chinese populations are tightly grouped together and located at the center of MDS plots, with the exception of one Han population sampled from Henan Province. We can also find that several minority groups, including Yunnan Bai, Xinjiang Xibe, Hubei Tujia and Aksu Uyghur, are intermingled with aforementioned Han Chinese groups. As expected, other 13 minority ethnic populations are scattered located in the MDS: Hainan Li is located in the top left corner; Sichuan Yi and Inner Mongolia Mongolian in the top right corner; Fujian She, Yunnan Yi, Inner Mongolia Mongolian and Qinghai Salar are being assigned in the lower left quarter; Ili Kazakh, Gansu Yugu, Lhasa Tibetan and Sichuan Tibetan are clustered together and located in the bottom right corner. Our new investigated Sichuan Tibetan keeps a close position with Lhasa Tibetan as our expectation, however, Sichuan Yi shows a subtle separation with Yunnan Yi.

### Phylogenetic relationship reconstruction

**Figure 4:**
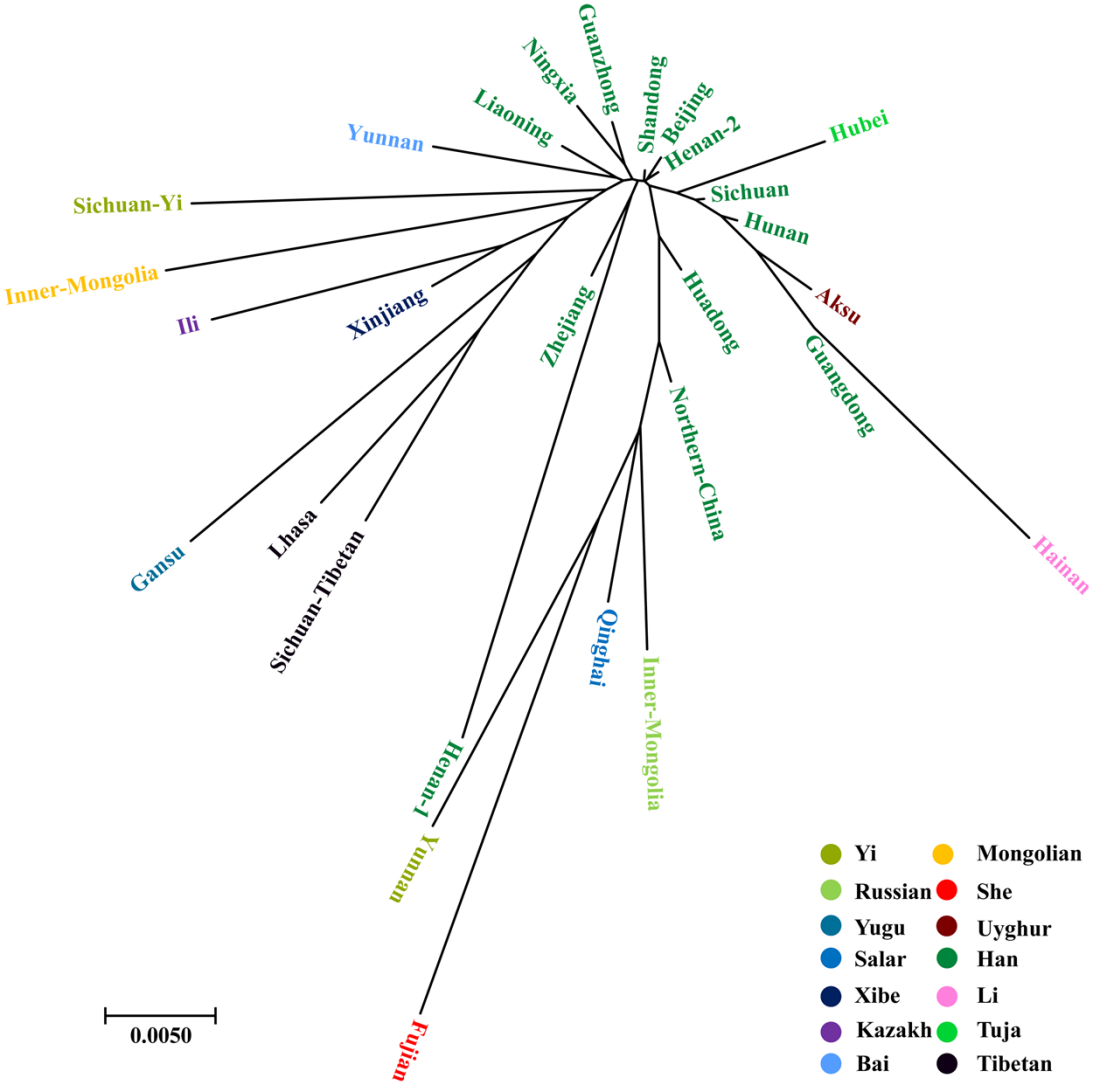
The Neighboring-Joining tree is prepared on the basis of the pairwise Nei’s genetic distance. The Phylogenetic tree constructed by a Neighbor-Joining algorithm method showing the genetic relationship of our two studied populations and additional 26 reference populations based on 21 non-CODIS STR markers. Each color label corresponding to ethnicity.

To further assess the Chinese population affiliations, we next sought to explore the phylogenetic relationship between Han Chinese populations and minority ethnic groups. The population relationships among 28 groups are depicted using a Neighbor-Joining tree based on Nei’s genetic distance matrix. As presented in Fig. 4, the Chinese Han populations are substantially differentiated from most of minority ethnic groups, especially significant in Chinese Muslim groups, Tibet high-altitude population, and even She ethnicity. Three main clusters can be clearly identified in the phylogenetic tree, the corresponding population compositions and distributions are congruent with the findings in the MDS and PCA. Notably, Sichuan Tibetan keeps genetic affinity with Lhasa Tibetan and first group together, and then cluster with Gansu Yugu population. Sichuan Yi is first clustered with Inner Mongolia Mongolian and then grouped with branch consisted of Liaoning Han and Yunnan Bai.

## Discussion

China is currently populated by over 1.3 billion people who belong to at least 56 Chinese officially recognized linguistically and ethnically different groups. Genetic studies of Chinese populations from different minority ethnic groups are of great interest due to China’s complex demographics, large population size and complex geographical characteristics. Additionally, clearly identifying and detecting the modern human evolution, origin and demographic history have been a resurgence of interest in population geneticists and medical geneticists due to successfully analyze the ancient nuclear and mitochondrial DNA sequences of Neanderthal and Denisovan^38–40^ and corresponding affection of anatomically modern human or present-day population disease susceptibility^41^. Previous anthropological and genetic studies have provided evidences that the peopling of China is characterized by different ancestral ethnicity origin by maternal lineages (mitochondrial genome) and paternal genetic signatures (Y-Chromosome)^42–48^. Despite these large-scale efforts in investigating patterns of natural selection, estimating individual ancestry and predicting the evolutionary history in Chinese populations based on SNPs, InDels, and CODIS STR loci^21–23,49,50^. The human genetic variation of non-CODIS STRs in the Sichuan Yi and Tibetan populations remains unexplored. In this study, a total of 237 Chinese Tibetan individuals and 95 Yi individuals from Southwest China are genotyped using AGCU 21 + 1 PCR amplification kit. In addition, the population differentiation analyses also included 9,444 individuals in 26 groups from 23 distinct administrative divisions or 14 ethnic groups that are genotyped using this same kit in the previous studies. The final data set is made up of 21 non-CODIS STR loci genotypes in 9,776 individuals from 28 Chinese populations. We have analyzed the genetic variation and population structure of the aforementioned populations via analysis of molecular variance, PCA, MDS and phylogenetic analyses.

### Forensic features of non-CODIS STRs in Tibetan and Yi

Recently, large number of commercial kits included the overlapped 13 CODIS STRs, such as GlobalFiler^TM^ STR kit^51^ (Thermo Fisher Scientific, Carlsbad, USA), Huaxia^TM^ Platinum PCR Amplification kit^52^ (Thermo Fisher Scientific, Carlsbad, USA), PowerPlex^®^ Fusion kit^53^ (Promega, USA) and so on, are widely used in the forensic human identification, paternity testing, and DNA database construction in criminal investigations or missing persons cases. CODIS STRs amplification systems with the limitation of improving the forensic efficiency when used them as a complementary to each other in the complex forensic cases. However, simultaneously testing the 21 non-CODIS STRs included in the AGCU 21 + 1 kit can minimize adventitious matches, increase discrimination power and facilitate data sharing in the cases with mutation, degraded sample cases and deficiency cases of paternity testing. Several measures of genetic diversity (observed heterozygosity, expected heterozygosity) and forensic statistical indexes of 21 non-CODIS STRs (PD, PE, PIC and so on) are relatively high in Sichuan Yi and Sichuan Tibetan populations in the present study. Most previous genetic studies based on a set of SNPs or STRs located on sex Chromosome show similar results^18,19,54,55^. But some researchers illustrated the lack of enough combined power of discrimination (0.99999999995713) and power of exclusion (0.97746) in Yi ethnicity^56^. However, the CPEs in the new studied populations and previous studied Han group^24^ are over 0.99999 and the CPDs are larger than 0.9999999999999999999. Our findings in these two investigated populations in combination with the previous studied Sichuan Han group^24^ demonstrated that twenty-one non-CODIS STRs included in AGCU 21 + 1 PCR amplification kit are highly discriminative and informative in diverse ethnic populations residing in Sichuan Province, West China, and should be used as a complementary tool in complicated paternity cases (parentage relationship identification with mutation, historical human skeletal remains, missing persons investigation and disaster victim identification). Besides, it can also be integrated into the new panel examined using the massively parallel sequencing platform, such as Ion S5 XL and Illumina-Miseq sequencer. This study also provides the first batch of genetic diversity information of 21 non-CODIS STRs in two ethnic groups and enriches the Chinese non-CODIS STRs reference databases.

### Inner and inter population structure construction

The results observed in this comprehensive population comparison reveal that significant genetic differences are identified between Han Chinese populations and some minority ethnic groups, especially predominantly in Tibetan and She populations. Besides, our analyses of phylogenetic relationship reconstruction and MDS indicated that Han Chinese population is homogenous based on autosomal genetic makers compared with sex-inherited genetic markers^57^. Among Han Chinese populations, no significant differences are observed in different populations defined by geographic boundary (Yangzi River), which is identified by the Y-STRs and high density SNPs panel^57^. However, a slight North-South gradient difference can be vaguely identified and not a significant North-South genetic distinction. The identified genetic similarities and differences among Chinese populations are a valuable technique for identifying accurate disease risk gene in genome-wide association study, avoiding a spurious association, and detecting more ethnicity-special ancestry informative markers in forensic ancestry inference.

Tibetan and Yi populations belong to the Tibeto-Burman-speaking subfamily in the Sino-Tibetan languages and the previously investigated Southwest Chinese Han population belongs to another subfamily (Chinese). Tibetan, as a most representative group, is genetically adapted to extreme hypoxia, and has been the genetic subject for multidisciplinary Studies. Our results reveal that two Tibetan populations distributed in different geographic positions (high altitude: Tibet, and low altitude: Sichuan) have a strong genetic affinity, however, keep a far genetic relationship with other populations. These features are consistent with previous findings revealed by genetic studies based on high-throughput genotyping data and genome sequence data^22,50,58^. Yi population, as we expected, keeps a relatively distant genetic relationship with the Tibetan population residing in Sichuan. Besides, these two Tibeto-Burman-speaking populations keep a relatively genetically distinct relationship with our investigated Sichuan Han population^24^ although all three groups have a close geographical position, which is accordance with different ethnicity origin, cultural background. It is strange to find that Sichuan Yi and Yunnan Yi keep a distant genetic relationship which may be influenced by the culturally different of three subgroups of Yi (Ni, Lolo, and other). In the future, large-scale population genetic history studies from different administrative divisions based on different high-density genetic marker sets (even whole genome sequence of archaic or present-day human DNA) will be needed to investigate and elucidate the origin, migration of the Sino-Tibetan-speaking ethnicity groups.

## Conclusions

In summary, we sampled 332 individuals from two minority ethnic groups to assess the genetic variations of 21 non-CODIS STR loci and combined these samples with 9,444 individuals previously investigated from 26 Chinese populations to explore Chinese population structures. Our results demonstrated that this panel of STRs is highly informative and polymorphic in the Sichuan Tibetan and Yi population, and can be widely used as a tool for personal identification and parentage testing in forensics. Additionally, the estimate of genetic differentiation (Fst and p values, and Nei’s genetic distance) suggested that the Sichuan Tibetan population and Sichuan Yi keep the close relationship with Lhasa Tibetan and Mongolian population, respectively, but being relatively isolated from other ethnic groups, especially within Han Chinese populations. The results obtained from PCA, MDS and phylogenetic analyses also demonstrated that genetic differences among Han Chinese and minorities widely exist and Han Chinese populations are homogeneous in different geographical divisions.

## Methods

### Ethnics standard

This study was approved by the institutional review boards of Sichuan University. All participants signed informed consent statements prior to participation. Human blood samples were collected upon approval of the Ethics Committee at the Institute of Forensic Medicine, Sichuan University. All the experimental procedures and the methods for each procedure were carried out in accordance with the approved guidelines of the Institute of Forensic Medicine, Sichuan University.

### Sample preparation

Unrelated blood samples were collected from 237 unrelated Tibetan individuals (120 males and 117 females) recruited from Chengdu and 95 Yi individuals (55 males and 40 females) recruited from Liangshan Yi Autonomous Prefecture, Sichuan Province. All individuals had been required to be the indigenous inhabitants or with a recent ancestor residing in the corresponding sample collection region at least three generations.

Human genomic DNA was extracted using PureLink Genomic DNA Mini Kit (Thermo Fisher Scientific) according to the manufacturer’s protocol. The quantity of the DNA template was determined using Quantifiler Human DNA Quantification Kit on a 7500 Real-time PCR System (Thermo Fisher Scientific). DNA samples were normalized to 1.0 ng/μL and stored at −20 °C until amplification.

### PCR amplification and genotyping

PCR amplification was performed following the manufacturer’s protocol on a ProFlex 96-well PCR System (Thermo Fisher Scientific). The PCR system was a 25 μL reaction volume containing 10 μL of Reaction Mix, 5 μL of Primers 21 + 1, 0.75 μL C-Taq and 1.0 ng of template DNA. The thermal cycling conditions consisted of an initial step at 95 °C for 2 min; followed by 30 cycles of 94 °C for 30 s, 60 °C for 60 s, and 65 °C for 90 s; and a final extension at 60 °C for 60 min.

Amplification products were separated and detected on the Applied Biosystems 3130 Genetic Analyzers following the manufacturer’s recommendations. One microliter of PCR products or allelic ladder was added to a mixture containing 9.5 μL of deionized Hi-Di formamide and 0.5 of μL AGCU Marker Size-500 (AGCU ScienTech Incorporation). The mixture was injected at 1.2 kV for 16 s and electrophoresed at 13 kV for 1550 s with a run temperature at 60 °C. Allele allocation was carried out with GeneMapper ID 3.20 analysis software using the allelic ladder and the set of bins and panels provided by the kit.

### Population studies

In order to evaluate the forensic efficiency of this non-CODIS STR panel for application in the Sichuan Tibetan and Yi, genotype data of 332 unrelated individuals were analyzed. The observed heterozygosity (Ho), expected heterozygosity (He), the exact test of Hardy-Weinberg equilibrium (HWE) and linkage disequilibrium (LD) were estimated and performed using Arlequin 3.5.2.2^59^. Allelic frequencies and forensic parameters, including the polymorphism information content (PIC), power of discrimination (PD), power of exclusion (PE) were calculated using the modified PowerStat V12 spreadsheet (Promega)^60^.

Additionally, 26 previously investigated populations^6–17,24–37^ comprising of 9444 individuals were retrieved to investigate the Sichuan and Chinese population genetic substructure. These reference populations include Salar (n = 120) residing in Qinghai Province, Tibetan (n = 104) from Lhasa in Tibetan autonomous region of Tibet, Tujia (n = 107) from Hubei Province, Bai (n = 106) from Yunnan Province, Han (n = 202) from Ningxia Hui Autonomous Region, Uyghur (n = 502) from Aksu region in Xinjiang Uyghur Autonomous Region, Kazakh (n = 114) from Ili region in Xinjiang Uyghur Autonomous Region, She (n = 154) from Fujian Province, Mongolian (n = 523) from Inner Mongolia Autonomous Region, Han (n = 220) from North China, Han (n = 275) from Guanzhong region in Shanxi Province, Yi (n = 110) from Yunnan Province, Li (n = 504) from Hainan Province, Han (n = 506) from Guangdong Province, Han (n = 501) from Hunan Province, Han (n = 225) from Huadong region of China, Han (n = 459) from Beijing Municipality, Han (n = 481) from Zhejiang Province, Russian (n = 114) from Inner Mongolia Autonomous Region, Han (n = 1030) from Shandong Province, Han (n = 207) from Liaoning Province, Han1 (n = 1136) from Henan Province, Yugu (n = 180) from Gansu Province, Xibe (n = 226) from Xinjiang Uyghur Autonomous Region, Han2 (n = 970) from Henan Province and Han (n = 368) from Sichuan Province. Population comparison was conducted using the principal components analysis (PCA), multidimensional scaling plots (MDS) and phylogenetic analysis. PCA was conducted using the Multivariate Statistical Package version 3.22^61^ and the SPSS software^62^ (version 21.0, SPSS Inc, Chicago, IL) based on the allele frequency distribution of 21 non-CODIS STRs in 28 Chinese populations. Genetic distance (Nei’s) was calculated using Phylip-3.695. The MDS was performed in SPSS and a Neighbor-Joining tree was constructed in Mega 7.0^63^ based on Nei’s genetic distance matrix^64^.

### Quality control

All experiments were conducted at the Forensic Genetics Laboratory of the Institute of Forensic Medicine, Sichuan University, which is an accredited laboratory (ISO 17025), and has been accredited by the China National Accreditation Service for Conformity Assessment (CNAS). We strictly followed the recommendations of Chinese National Standards and Scientific Working Group on DNA Analysis Methods (SWGDAM)^65^. Control DNA 9947A (AGCU ScienTech Incorporation) and ddH_2_O were used as positive and negative controls respectively for each batch of genotyping.

## Electronic supplementary material

Supplementary Figures S1-S4 and Supplementary Tables S1-S9
