# Supplementary material for: Genetic diversity and phylogenetic characteristics of Chinese Tibetan and Yi minority ethnic groups revealed by non-CODIS STR markers
Source: Sci Rep. 2018 Apr 12;8:5895. doi: 10.1038/s41598-018-24291-5 (PMC5897523; doi:10.1038/s41598-018-24291-5)
Supplement: Supplementary file 1 — Supplementary Figures S1-S4 and Supplementary Tables S1-S9 [file 41598_2018_24291_MOESM1_ESM.pdf]

Supplementary Figures S1-S4 and Supplementary Tables S1-S9

Genetic diversity and phylogenetic characteristics of Chinese Tibetan and Yi minority ethnic groups revealed by non-CODIS STR markers

Guanglin He[1,+], Zheng Wang[1,+], Xing Zou[2], Xu Chen[3], Jing Liu[1], Mengge Wang[1], Yiping Hou[1]*

[1]Institute of Forensic Medicine, West China School of Basic Medical Sciences & Forensic Medicine, Sichuan University, Chengdu, Sichuan, China

[2]Department of Forensic Medicine, College of Basic Medicine, Chongqing Medical University, Chongqing, China

[3]Department of Clinical Laboratory, the First People's Hospital of Liangshan Yi Autonomous Prefecture, Xichang, Sichuan, China

[+] These authors contributed equally to this work.

[*] Corresponding author: Yiping Hou

Institute of Forensic Medicine, West China School of Basic Medical Sciences & Forensic Medicine, Sichuan University, Chengdu 610041, China

E-mail: profhou@yahoo.com; Phone: +86-28-85501550; Fax: +86-28-85501549.

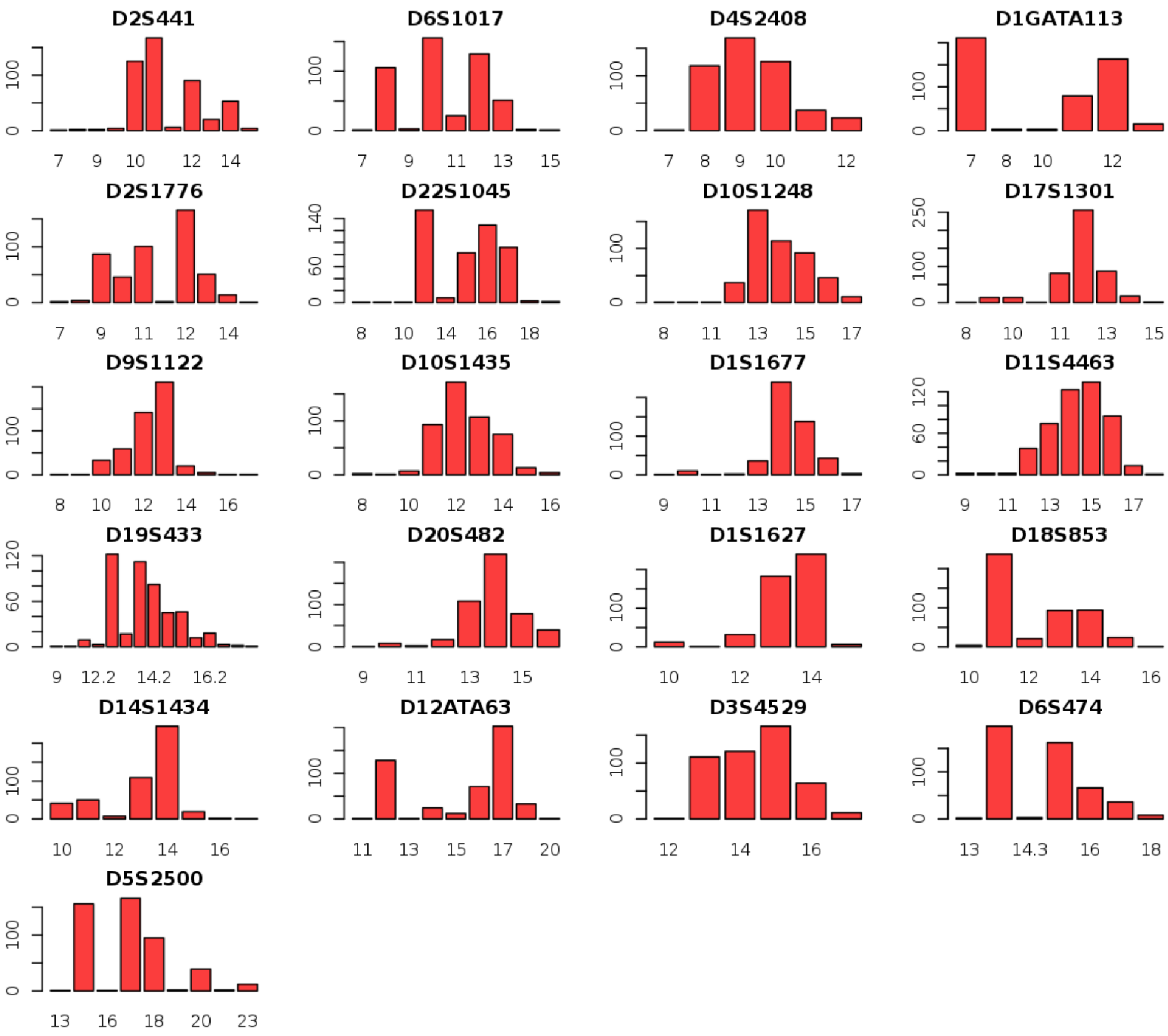

Supplementary Figure S1. Plots allele frequency distributions of 21 non-CODIS STRs in the Sichuan Tibetan population.

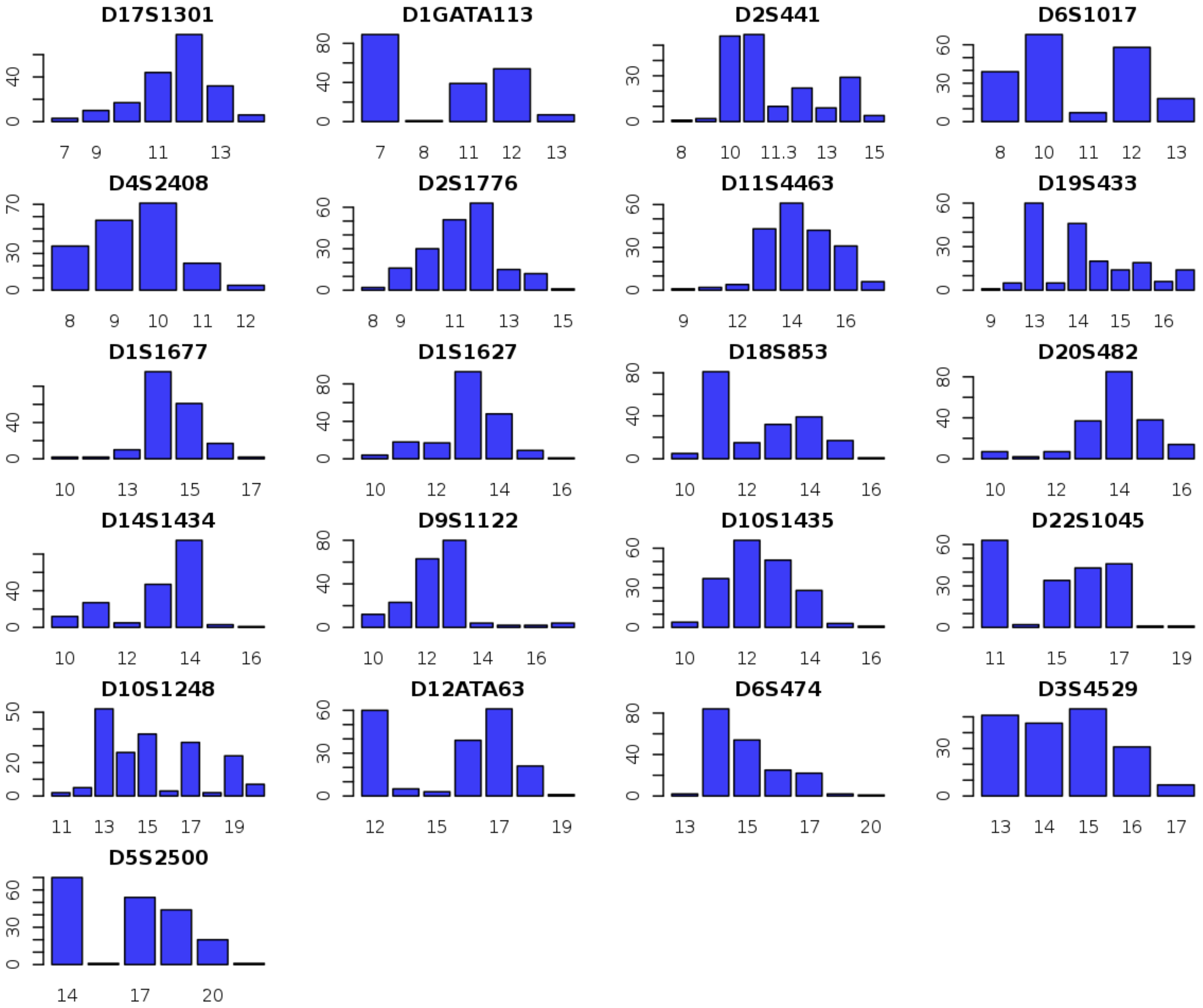

Supplementary Figure S1. Plots allele frequency distributions of 21 non-CODIS STRs in the Sichuan Yi population.

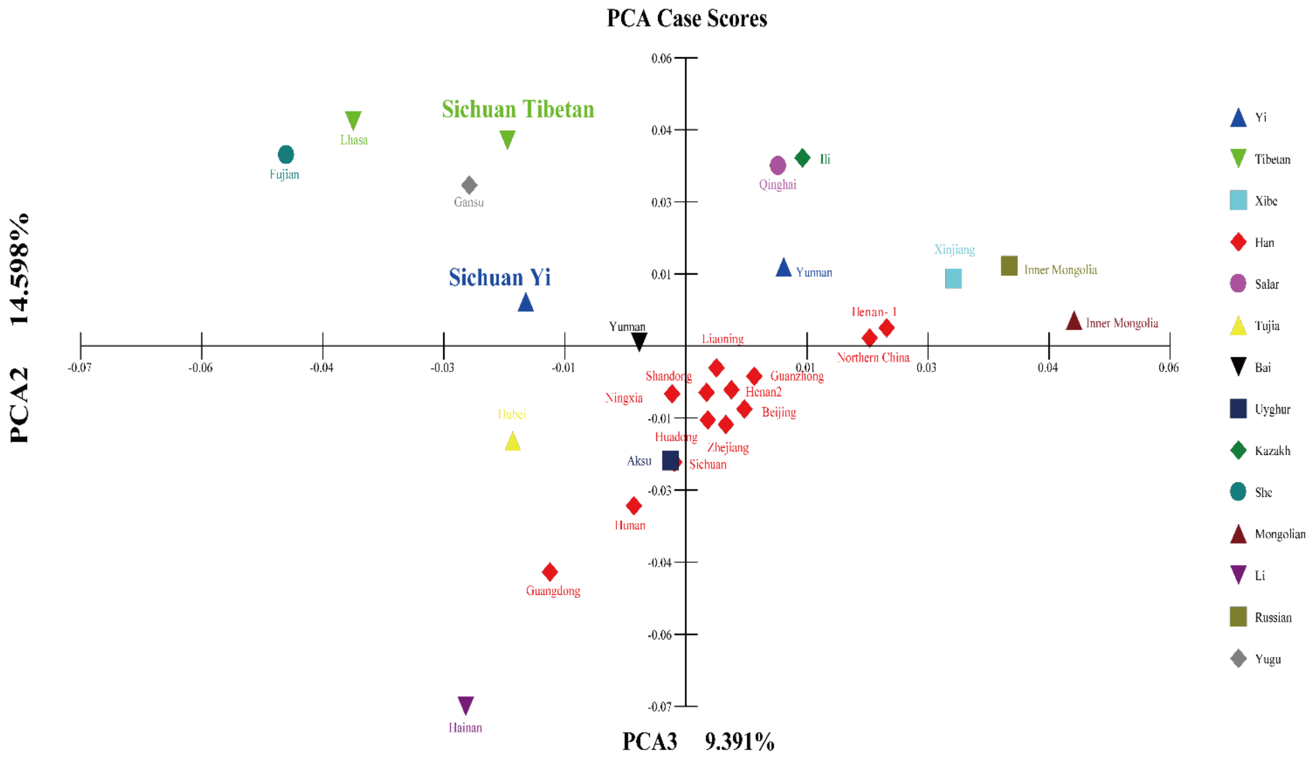

Supplementary Figure S3. The principle component analysis showed the genetic relationship of Sichuan Tibetan, Sichuan Yi and 26 Chinese populations based on the second and third components.

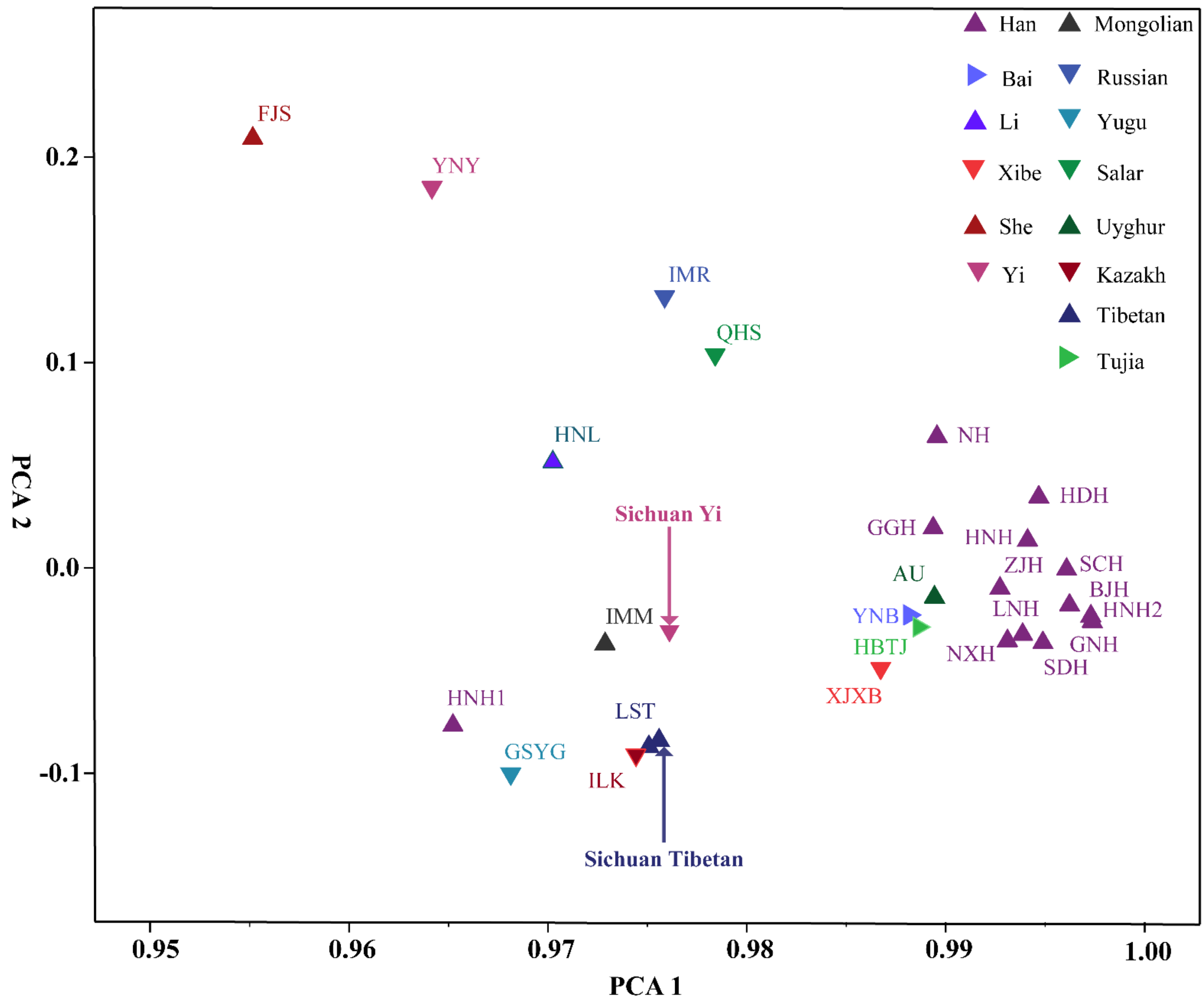

Supplementary Figure S4. The principal component analysis (PCA) of population structure among 28 Chinese populations was constructed by the SPSS. The results displayed population relationships between the Sichuan Tibetan, Sichuan Yi populations and other 26 reference populations based on the variances of the first, second components. Each population is represented by one triangle and the color label corresponding to ethnicity (LST: Lhasa-Tibetan; QHS: Qinghai-Salar; HBTJ: Hubei-Tujia; YNB: Yunnan-Bai; NXH: Ningxia-Han; AU: Kuqa-Uyghur; ILK: Ili-Kazakh; FJS: Fujian-She; IMM: Inner-Mongolia-Mongolian; NH: Northern-Han; GNH: Guanzhong-Han; YNY: Yunnan-Yi; HNL: Hainan-Li; GGH: Guangdong-Han; HNH: Hunan-Han; HDH: Huadong-Han; BJH: Beijing-Han; ZJH: Zhejiang-Han; IMR: Inner-Mongolia-Russian; SDH: Shandong-Han; LNH: Liaoning-Han; HNH2: Henan-Han-2; GSYG: Gansu-Yugu; XJXB: Xinjiang-Xibe; HNH1: Henan-Han-1; SCH: Sichuan-Han).

Supplementary Table S1. The genotype of 237 Tibetan individuals residing in the Chengdu city in Sichuan Province, Southwest China

Num	Amelo	D6S	D12A	D22S	D10S	D1S1	D11S	D1S1	D3S4	D2S	D6S1	D4S2	D19S	D17S	D1GAT	D18S	D20S	D14S	D9S1	D2S1	D10S	D5S2
ber	genin	474	TA63	1045	1248	677	4463	627	529	441	017	408	433	1301	A113	853	482	1434	122	776	1435	500
N00	X	16	14	16	12	13	15	13	14	12	8	10	13	12	7	11	13	13	11	12	13	17
1	X	17	17	17	14	14	16	13	16	14	10	10	13	13	12	14	15	14	11	12	14	18
N00	X	14	12	16	15	14	13	12	15	11	8	8	13	9	11	11	14	13	13	10	12	14
2	X	16	12	17	17	16	15	13	15	12	10	9	15.2	12	12	11	15	13	14	12	14	17
N00	X	14	14	15	14	15	14	14	15	10	8	9	14	12	7	11	11	13	10	10	13	14
3	X	14	17	15	15	15	16	14	17	12	12	10	14	12	12	11	14	14	10	11	13	17
N00	X	14	15	11	13	15	14	14	14	10	8	8	15.2	11	7	11	13	14	10	9	13	20
4	X	15	17	16	16	16	14	14	16	14	13	10	15.2	12	7	14	14	14	13	12	14	23
N00	X	14	12	11	12	14	15	13	13	10	8	8	13.2	12	7	11	14	13	11	12	12	14
5	X	14	16	11	14	15	16	13	15	14	12	10	14.2	13	7	11	14	13	11	14	13	17
N00	X	14	17	11	15	15	13	14	15	12	10	8	14.2	11	12	11	13	14	10	10	8	14
6	X	14	17	11	17	15	14	15	16	14	12	9	15	12	12	13	14	15	13	12	12	18
N00	X	14	12	17	14	16	17	13	15	11	9	9	15.2	11	7	12	14	15	12	12	14	16
7	X	14	15	18	14	16	18	13	15	12	11	10	18	13	12	12	15	16	13	14	15	21
N00	X	14	17	16	14	14	13	12	14	10	10	9	12.2	12	7	11	14	14	12	9	13	14
8	X	15	18	17	15	14	16	14	16	14	12	10	13	12	12	14	14	14	13	12	14	14
N00	X	15	17	11	14	14	15	13	13	11	10	8	13	12	7	11	12	11	10	9	11	14
9	X	16	17	16	14	14	16	13	14	12	13	10	13	12	7	11	14	14	13	13	12	17
N01	X	15	12	11	12	14	12	12	13	11	12	9	12	12	7	14	13	10	11	9	12	14
0	X	15	17	16	13	15	15	13	14	15	12	9	16.2	12	11	14	14	13	11	12	14	14
N01	X	15	14	16	13	14	15	13	15	11	12	9	13	12	7	11	15	13	11	12	11	14
1	X	15	17	17	15	14	15	13	16	12	13	10	17.2	12	12	13	15	14	13	13	12	20
N01	X	14	14	16	13	14	13	13	13	11	12	9	13	12	7	13	14	14	11	12	12	17
2	X	15	17	17	15	14	15	13	15	12	12	10	14	12	12	14	15	14	13	12	12	17
N01	X	14	17	14	13	15	12	14	14	11	10	9	12	12	7	11	14	13	10	9	11	14
3	X	15	18	15	13	17	15	14	14	11	13	11	13	12	12	13	14	14	11	12	11	14
N01	X	15	17	15	13	15	15	13	15	11	8	8	13	11	7	11	13	10	13	9	11	20
4	X	15	17	17	13	15	16	14	15	13	10	10	14.2	12	7	11	14	13	13	12	12	23

N01	X	15	17	16	13	14	12	13	13	10	8	10	14	12	11	11	13	13	12	12	13	17
5	X	17	17	17	13	15	12	14	14	12	8	12	14.2	14	12	14	14	14	13	12	14	20
N01	X	14	12	11	13	15	13	14	15	10	8	8	14.2	11	7	11	13	13	12	9	11	17
6	X	15	17	15	15	16	15	14	15	11	12	9	14.2	12	12	13	15	14	13	14	13	20
N01	X	14	17	15	12	14	16	14	16	10	8	8	13	12	7	11	13	13	12	12	11	20
7	X	14	17	16	14	15	16	14	16	11	12	10	13	12	7	13	13	14	13	13	14	23
N01	X	14	16	16	15	13	13	14	13	12	10	8	13	12	7	11	14	10	11	12	11	14
8	X	15	17	17	16	15	15	14	14	13	10	8	14.2	12	13	14	15	13	12	12	12	14
N01	X	14	15	11	13	14	14	14	15	10	11	8	14.2	11	7	14	13	13	12	12	13	14
9	X	14	18	11	17	15	16	14	15	12	12	10	14.2	12	7	14	14	13	13	12	14	18
N02	X	15	12	11	13	15	12	14	13	10	11	9	14	13	12	11	13	14	12	9	11	14
0	X	16	14	11	14	16	17	14	15	12	13	10	14.2	13	12	12	16	14	12	12	12	18
N02	X	14	17	11	12	10	14	13	13	10	10	10	14	12	7	11	15	12	11	9	12	17
1	X	16	17	15	16	14	15	14	13	11	12	12	15	13	7	11	15	14	12	9	12	20
N02	X	14	17	15	13	14	12	14	15	10	13	9	14	12	7	13	13	14	12	10	12	14
2	X	15	18	16	16	16	13	14	15	14	13	10	14.2	12	12	15	14	14	13	12	12	17
N02	X	16	17	11	12	14	15	14	13	10	10	8	14	12	7	11	13	11	12	12	13	17
3	X	17	17	11	16	14	15	14	15	11	13	9	14.2	12	11	15	14	14	13	13	14	23
N02	X	14	17	16	13	15	12	14	13	12	8	9	13	11	11	13	13	14	12	9	12	14
4	X	15	17	17	15	16	15	14	15	14	13	10	14.2	12	12	13	14	14	13	10	12	14
N02	X	15	17	11	13	15	14	14	15	10	12	8	15	12	7	11	14	14	12	9	13	14
5	X	16	17	16	14	16	14	14	15	12	12	10	15.2	14	7	14	15	14	13	12	14	20
N02	X	14	16	16	13	15	12	14	15	11	8	8	13	10	7	13	13	13	11	11	11	14
6	X	17	17	17	14	16	16	14	15	12	12	9	15	12	11	13	14	14	13	15	13	23
N02	X	14	12	11	15	15	13	14	15	10	8	9	14	12	7	11	13	14	12	10	13	17
7	X	15	12	11	16	16	14	14	16	11	12	12	15	12	12	13	14	14	12	11	16	17
N02	X	15	18	16	14	14	14	14	13	10	8	9	13	11	7	11	14	11	12	11	11	20
8	X	17	18	17	14	15	15	14	16	11	10	10	16	12	7	14	16	14	14	14	13	20
N02	X	14	17	15	13	14	15	13	13	11	8	8	13	12	7	11	14	13	10	10	11	17
9	X	14	17	17	15	14	16	14	15	12	12	9	14	12	12	14	14	14	13	12	12	17
	X	15	12	15	13	14	14	13	13	10	10	8	13.2	12	12	11	16	10	10	9	12	17

N03																						
0	X	15	17	16	15	14	14	14	16	11	11	12	15	13	12	12	16	13	12	12	14	18
N03	X	14	17	11	12	14	13	13	14	10	8	8	13	11	7	13	14	14	13	12	12	17
1	X	18	17	15	13	15	13	14	16	11	10	12	15.2	14	12	15	14	14	13	12	13	20
N03	X	14	12	15	15	14	13	13	13	11	12	8	13	11	7	11	14	14	11	9	11	17
2	X	14	18	16	16	14	14	14	14	11	13	9	14	13	12	14	15	14	12	13	13	17
N03	X	14	16	11	13	14	12	10	13	10	10	8	12	12	11	11	14	14	10	11	14	14
3	X	14	17	15	14	16	15	14	14	12	12	8	14.2	12	12	11	14	14	12	12	14	17
N03	X	17	12	14	14	14	14	13	13	12	8	9	15	12	7	13	13	13	12	11	12	17
4	X	17	17	16	14	14	15	14	13	12	13	10	15.2	13	11	14	14	14	13	13	12	18
N03	X	15	15	11	14	10	15	13	14	11	8	8	13	11	7	11	13	14	13	9	12	18
5	X	15	17	15	15	14	16	14	14	11	10	8	13	12	12	11	14	14	14	11	13	20
N03	X	14	16	15	13	14	14	13	14	11	10	9	13	11	7	11	13	13	11	9	13	14
6	X	14	18	17	16	14	16	14	17	13	10	10	13.2	12	7	13	14	14	12	12	13	18
N03	X	14	12	15	13	13	13	13	15	10	8	8	13.2	11	7	13	13	13	12	12	11	18
7	X	17	14	16	16	14	14	14	15	10	12	9	14	13	12	13	14	14	13	13	15	23
N03	X	15	16	11	13	14	15	13	13	10	10	9	13.2	11	11	11	13	13	10	10	11	14
8	X	16	18	11	14	15	16	14	15	12	11	12	16.2	12	11	14	15	14	13	11	14	17
N03	X	14	12	11	15	14	14	13	15	10	8	9	13	11	12	11	13	14	12	10	13	14
9	X	14	17	16	15	15	14	13	17	11	12	10	15	12	12	14	14	14	13	11	13	18
N04	X	14	17	11	13	14	15	13	15	10	10	8	13	10	11	13	13	10	12	10	12	14
0	X	14	17	15	14	16	15	14	16	12	12	10	14.2	12	12	14	13	14	13	12	12	17
N04	X	15	12	11	13	14	16	14	14	11	10	9	13	11	7	13	13	13	13	12	13	14
1	X	17	12	11	14	14	16	14	16	12	12	10	15	12	11	14	13	14	13	12	14	18
N04	X	14	17	15	13	13	12	10	13	10	10	8	13	9	7	13	14	14	12	11	12	14
2	X	14	17	17	14	16	15	13	15	11	12	10	13	11	12	16	15	14	12	13	12	14
N04	X	15	17	15	13	14	14	13	13	9.1	10	8	14	11	7	11	16	14	13	9	11	14
3	X	16	17	17	13	14	16	14	14	11	10	9	14.2	11	11	11	16	14	13	10	11	18
N04	X	14	12	16	13	13	14	13	15	10	8	10	13	11	7	11	13	14	13	9	11	17
4	X	16	15	17	13	14	15	14	15	11	12	11	14	13	12	14	16	14	13	13	12	18
	X	14	17	15	13	13	14	13	13	12	10	9	13	12	7	11	16	13	13	11	12	14

N04																						
5	X	15	18	17	13	14	16	14	15	14	13	9	13.2	13	7	15	16	14	13	12	14	18
N04	X	16	17	11	13	15	14	13	15	11	8	8	13.2	13	7	13	13	14	12	11	12	17
6	X	16	17	11	15	15	15	14	16	12	12	8	14.2	14	11	14	14	14	12	13	13	17
N04	X	14	12	15	12	14	14	13	14	10	10	9	14.2	11	7	11	13	11	11	12	12	14
7	X	15	17	16	16	14	15	13	16	14	11	9	15	12	8	11	14	15	13	13	13	17
N04	X	14	16	16	13	13	15	13	14	10	8	9	14	11	7	11	13	13	12	12	12	17
8	X	14	17	17	13	14	15	14	16	12	8	11	16.2	13	11	11	14	14	13	12	12	18
N04	X	14	12	11	14	14	13	13	14	10	8	8	13	12	7	11	14	14	14	11	12	14
9	X	14	12	16	14	14	15	14	17	11	10	10	14.2	13	12	13	16	14	14	11	12	18
N05	X	14	12	11	13	14	13	13	15	10	10	9	13	12	7	11	14	10	11	9	12	14
0	X	14	17	15	14	14	14	13	16	11	12	9	13	15	11	11	16	10	12	12	16	18
N05	X	14	12	16	13	14	13	14	13	12	8	10	14.2	11	7	11	14	10	11	12	11	17
1	X	15	12	17	15	14	14	14	16	14	8	11	15	12	10	15	14	14	13	13	13	17
N05	X	17	12	11	15	14	13	13	15	11	8	8	13	10	7	11	13	14	12	12	11	14
2	X	18	16	11	16	14	16	13	17	14	8	8	14	11	11	14	14	14	13	12	12	18
N05	X	15	12	11	12	14	16	13	14	11	10	8	14	12	7	11	14	14	12	11	11	17
3	X	15	16	14	14	14	16	14	15	11	11	9	14	14	7	11	15	15	13	12	12	17
N05	X	14	12	11	14	13	14	13	14	15	12	10	14	12	11	11	15	13	10	9	11	17
4	X	14	16	16	14	14	16	14	14	15	13	10	14.2	12	12	13	15	14	12	9	14	18
N05	X	14	12	11	14	15	13	10	15	10	10	10	14	12	11	11	13	13	11	9	11	14
5	X	15	12	11	14	16	13	14	15	12	12	10	16.2	12	11	11	13	14	13	12	12	14
N05	X	15	12	15	13	13	14	13	15	11	8	9	14.2	13	7	11	15	13	13	12	11	14
6	X	15	17	16	15	14	15	15	16	13	12	10	15	13	7	14	15	13	13	13	12	18
N05	X	14	12	11	13	14	14	12	13	10	12	8	14.2	11	12	12	14	10	11	9	12	17
7	X	15	17	11	15	14	16	14	16	10	12	10	15	12	12	13	14	13	13	13	12	17
N05	X	14	14	11	13	14	14	12	13	10	10	8	14	12	7	11	14	14	12	11	11	17
8	X	14	16	11	13	16	15	14	14	11.3	12	8	16.2	13	11	13	14	14	13	12	15	17
N05	X	14	17	15	14	13	12	12	14	12	13	9	14	12	7	11	14	13	11	9	12	14
9	X	14	17	16	15	14	13	13	15	13	13	11	14	12	12	11	15	14	12	12	14	17
	X	14	16	16	12	14	12	13	15	10	10	8	13	12	11	11	13	13	11	9	11	17

N06																						
0	X	14	17	16	13	16	15	14	15	12	11	9	14.2	14	12	13	14	14	13	9	12	18
N06	X	14	15	17	12	13	12	13	13	11	8	9	14	9	12	11	14	13	13	9	12	17
1	X	14	18	18	15	14	13	14	14	11	12	10	14.2	12	12	14	15	14	13	11	13	18
N06	X	15	12	15	13	15	14	13	13	11	10	9	14	12	7	13	13	10	12	9	12	17
2	X	18	16	16	16	15	14	14	14	14	10	11	15	12	7	13	15	14	13	12	12	17
N06	X	15	16	11	13	14	14	14	14	11	8	9	13	10	7	11	14	13	13	12	11	14
3	X	15	17	18	15	14	16	14	15	11	12	10	14	10	11	11	14	13	13	13	11	14
N06	X	14	17	15	15	14	14	13	13	11	10	10	14.2	12	7	11	13	13	13	11	11	17
4	X	15	18	16	15	15	16	14	15	13	10	10	17	14	12	14	14	14	14	12	12	17
N06	X	15	12	11	13	16	15	13	13	10	10	8	14	12	7	10	14	11	12	12	11	14
5	X	17	12	16	17	16	16	15	15	12	13	11	15	13	12	14	14	13	14	13	12	17
N06	X	15	16	11	14	14	14	13	16	10	10	9	14	11	7	11	13	14	13	12	11	14
6	X	16	18	16	14	15	15	14	16	11	10	9	15	12	12	14	14	14	13	13	12	17
N06	X	14	12	11	13	14	15	13	13	11	10	9	13	12	12	11	14	11	12	9	12	18
7	X	15	17	17	16	14	16	14	14	11	10	11	16	13	13	11	14	13	12	11	14	20
N06	X	14	14	11	12	14	14	12	13	11	12	10	13	11	7	11	13	13	11	9	13	17
8	X	15	17	16	13	15	15	13	14	12	12	12	13	13	11	14	14	14	13	13	13	20
N06	X	14	12	11	13	14	12	12	15	12	8	9	14	12	12	11	13	13	14	9	11	14
9	X	14	17	11	14	15	14	13	15	14	10	9	15.2	14	12	11	14	14	16	11	13	17
N07	X	15	16	15	13	14	14	13	14	10	8	8	15.2	11	11	11	13	13	12	9	11	17
0	X	16	17	17	13	14	15	13	15	12	13	10	16	13	12	14	15	14	13	14	13	18
N07	X	14	14	16	13	14	12	13	13	11	8	9	14.2	11	7	11	14	14	11	13	12	14
1	X	14	17	16	14	14	15	13	14	11	13	11	17.2	13	7	14	14	14	12	13	13	14
N07	X	14	12	16	13	14	14	13	14	11	9	8	13	12	12	11	14	13	12	9	14	17
2	X	15	17	16	13	14	15	14	15	11	13	9	15	12	12	13	14	14	13	12	14	17
N07	X	14	16	15	15	14	14	13	13	10	12	8	14	12	7	13	13	14	11	11	12	17
3	X	14	17	17	16	14	15	14	14	11	13	9	14.2	12	11	15	14	15	11	13	14	17
N07	X	15	16	16	14	14	15	13	13	11	8	10	13	11	12	11	10	11	13	9	12	14
4	X	16	17	17	16	15	17	14	13	12	10	11	13	11	12	14	13	14	13	12	14	14
	X	14	12	16	14	14	13	13	15	11	10	8	13	12	7	11	14	13	12	7	12	14

N07																						
5	X	17	17	16	16	15	15	14	15	11	12	10	13.2	13	11	14	15	14	13	12	13	18
N07	X	15	17	10	13	15	15	13	13	11	13	11	14	12	7	11	14	13	11	8	12	17
6	X	15	18	17	15	15	16	14	14	14	13	11	15	12	7	11	14	13	13	12	13	18
N07	X	14	14	16	13	14	14	14	14	11	10	9	13	12	11	11	13	10	12	9	13	14
7	X	15	17	16	15	14	16	14	15	11	10	10	15.2	12	12	14	14	14	13	13	13	17
N07	X	14	17	11	13	10	13	14	15	11.3	8	8	12	11	7	11	14	13	11	9	13	17
8	X	16	17	17	14	13	14	14	15	12	12	10	14	13	13	12	14	14	14	12	14	18
N07	X	14	16	11	12	14	12	14	15	11	8	9	13	10	11	11	14	13	11	11	11	14
9	X	15	17	14	15	15	14	14	16	12	12	10	14.2	13	12	11	14	14	13	11	11	20
N08	X	15	12	16	12	14	14	13	15	10	10	9	12	11	11	14	12	14	12	11	13	14
0	X	15	17	17	14	14	15	14	16	10	12	9	14	11	11	14	14	14	13	12	13	14
N08	X	15	12	17	13	15	14	14	13	10	10	9	14	12	12	11	13	10	12	12	12	14
1	X	17	16	17	14	15	14	14	15	11	12	10	14	13	12	13	14	14	13	12	14	17
N08	X	14	16	17	13	14	13	13	13	13	10	8	14	11	11	11	13	11	12	11	13	17
2	X	15	18	17	16	14	14	14	14	14	10	8	14.2	12	13	14	14	14	13	13	14	20
N08	X	14	12	11	15	10	15	14	13	10	8	8	14	11	7	11	15	12	11	9	11	17
3	X	16	17	15	16	16	16	14	13	12	8	9	14	13	12	11	16	14	13	11	12	17
N08	X	15	12	15	13	13	9	13	14	10	10	9	15	12	7	11	12	13	10	11	11	14
4	X	15	17	17	13	16	16	13	15	11	13	10	15.2	13	7	13	14	14	14	12	12	18
N08	X	14	12	11	12	12	16	14	15	10	11	9	13	12	7	11	14	10	12	12	11	14
5	X	16	17	11	15	14	17	14	17	10	13	10	15	12	7	14	16	11	13	12	14	17
N08	X	14	18	15	15	14	15	10	15	10	8	9	13	12	7	11	14	13	12	12	12	18
6	X	15	18	16	16	14	16	13	15	13	10	10	16.2	13	7	11	14	14	13	14	15	18
N08	X	14	12	11	13	14	13	12	14	11	12	8	14	13	7	11	14	14	12	9	12	14
7	X	15	17	16	15	15	14	12	15	14	12	10	15.2	13	12	15	14	14	13	10	14	18
N08	X	14	12	17	13	14	15	13	15	11	8	9	13	12	7	11	15	11	10	9	12	18
8	X	16	17	17	16	15	16	14	15	12	12	12	14.2	13	11	11	16	15	13	10	14	20
N08	X	14	16	11	12	14	14	13	13	11	12	8	14	11	7	12	14	10	11	11	13	14
9	X	15	17	11	16	14	15	14	13	11	12	9	14.2	12	11	13	14	14	12	12	14	18
	X	14	17	16	13	14	14	13	13	11	8	8	13	12	11	11	14	14	12	10	12	17

N09																						
0	X	15	17	17	13	15	14	13	15	13	13	8	14	12	12	11	14	14	13	12	13	21
N09	X	16	16	15	13	13	14	14	13	11	12	9	13	12	7	13	14	13	11	11	13	14
1	X	17	17	17	15	14	15	14	14	11	12	9	14.2	13	12	14	15	14	13	11	13	17
N09	X	16	12	11	12	13	13	13	15	11	10	8	12	12	7	11	13	13	13	10	12	18
2	X	16	16	15	13	15	14	13	17	12	13	9	15	12	12	15	16	14	13	13	12	18
N09	X	15	12	15	13	15	15	13	14	12	10	9	12	9	11	11	15	13	13	11	12	17
3	X	16	17	16	14	16	15	14	15	12	12	11	14	12	12	13	16	15	13	12	13	18
N09	X	14	12	14	13	10	15	14	13	10	8	10	14.2	12	7	11	14	13	12	12	11	17
4	X	14	17	16	14	16	16	14	13	10	10	11	15	13	12	13	14	14	13	12	11	18
N09	X	15	12	11	14	14	14	14	14	11	10	8	14	12	12	11	14	11	13	9	11	14
5	X	15	16	17	14	14	14	14	15	14	10	10	15.2	12	12	14	15	12	14	11	12	17
N09	X	14	16	16	13	14	14	14	15	10	10	9	13	10	11	11	13	13	13	12	13	14
6	X	17	17	17	14	15	15	14	16	10	12	11	14	13	13	14	13	14	13	12	14	17
N09	X	15	17	11	14	14	15	14	13	10	10	8	13	11	7	13	14	11	12	10	11	14
7	X	15	17	16	16	16	17	14	14	11	11	11	16.2	12	12	14	16	14	13	11	12	17
N09	X	14	12	16	15	14	13	13	14	10	12	9	14.2	11	7	11	14	11	10	9	12	18
8	X	16	16	16	16	14	13	13	14	10	12	10	15	12	12	14	15	14	13	9	15	18
N09	X	14	15	11	13	15	14	13	15	11	8	8	14	12	11	11	13	14	12	11	11	17
9	X	15	17	16	14	15	16	13	16	11	11	11	15	12	12	14	14	14	12	11	14	18
N10	X	14	12	11	13	15	13	14	13	12	8	9	14	12	11	11	13	14	11	10	13	14
0	X	17	14	17	13	15	15	14	14	12	12	9	15	13	12	14	14	14	12	12	14	14
N10	X	14	15	15	14	14	13	12	13	9	10	9	13	12	7	11	13	14	13	11	11	14
1	X	16	16	16	15	14	15	13	15	10	12	9	14	12	12	15	14	14	14	12	12	17
N10	X	14	17	11	13	14	12	13	14	11	10	9	13	12	11	11	13	14	12	10	11	14
2	X	17	17	11	14	15	15	14	15	14	12	11	13	12	11	11	13	14	13	10	13	20
N10	X	14	17	11	13	14	13	13	13	10	8	9	13	11	12	13	13	14	12	12	13	17
3	X	14	17	17	14	15	14	14	15	14	8	9	16.2	13	13	14	16	14	13	13	13	17
N10	X	14	16	16	12	14	15	12	13	10	10	10	14	12	7	11	12	10	12	10	11	17
4	X	15	17	16	15	15	15	14	15	14	10	10	15	12	12	12	15	14	13	13	14	17
	X	14	12	16	13	15	12	13	14	10	8	8	13	13	7	11	12	14	13	10	12	14

N10																						
5	X	16	17	17	15	15	15	14	15	12	8	9	14.2	15	12	11	13	15	15	12	12	17
N10	X	15	17	11	13	13	13	13	13	11	10	10	14	11	7	11	10	11	13	10	12	14
6	X	15	17	16	14	15	14	14	14	14	10	11	15.2	12	12	13	16	13	14	11	12	18
N10	X	14	16	15	13	14	13	13	13	10	8	8	14.2	12	11	11	12	11	12	9	12	14
7	X	15	18	17	14	15	14	14	15	11	12	9	16	13	12	11	15	14	13	11	13	17
N10	X	14	12	11	14	14	14	13	16	11	10	8	15.2	11	7	11	14	11	10	11	12	17
8	X	15	17	16	16	15	14	14	16	12	13	11	16.2	13	12	11	15	13	12	14	14	23
N10	X	14	12	15	14	14	12	13	13	11	8	10	13	12	7	13	15	14	13	9	11	14
9	X	14	17	16	14	16	17	14	16	12	11	10	15.2	12	12	13	15	14	13	13	14	18
N11	X	14	12	15	13	13	14	13	14	11	10	10	13	12	7	13	14	11	11	11	14	14
0	X	17	16	16	13	15	14	14	16	12	12	10	14.2	12	13	14	14	11	11	12	14	17
N11	X	14	12	16	13	14	12	12	14	12	10	8	15.2	13	7	11	14	10	12	12	10	14
1	X	15	17	17	13	15	14	13	14	15	13	12	15.2	13	7	12	16	13	12	12	12	18
N11	X	15	12	11	13	14	15	12	14	10	10	9	13	12	7	11	15	10	12	9	13	17
2	X	16	12	17	13	14	16	12	15	14	10	10	13.2	12	12	15	15	11	13	11	14	19
N11	X	14	12	16	13	14	13	13	15	12	10	8	13	12	7	11	14	13	10	11	14	14
3	X	17	17	16	17	16	15	14	15	14	10	8	14	13	11	14	14	14	13	12	15	17
N11	X	14	16	11	12	14	14	13	14	9	10	8	13	11	7	11	13	13	13	9	11	14
4	X	14	17	16	14	15	16	14	15	10	10	9	15	12	12	13	16	14	13	13	12	17
N11	X	14	12	16	14	10	15	13	14	11	8	9	13.2	11	11	11	14	14	12	11	12	17
5	X	17	12	16	14	14	16	14	14	14	12	11	13.2	12	11	11	15	14	13	13	13	18
N11	X	14	15	17	13	14	13	14	13	10	8	9	14	12	7	13	14	11	12	11	13	17
6	X	15	17	17	14	15	16	14	15	10	12	10	14.2	12	11	14	15	14	13	11	16	18
N11	X	15	17	11	15	14	13	13	15	10	12	9	14.2	11	7	11	13	11	11	11	11	14
7	X	15	17	16	16	14	15	14	15	11	12	9	15.2	12	7	11	15	13	12	12	12	17
N11	X	13	11	8	8	9	9	10	12	7	7	7	9	8	7	10	9	10	8	7	8	13
8	Y	14	12	9	9	10	10	11	13	8	8	8	10	9	8	11	10	11	9	8	9	14
N11	X	15	12	16	13	14	14	13	15	11	10	10	13	11	7	11	14	13	11	11	13	14
9	Y	15	17	17	15	14	16	14	15	12	10	11	14.2	12	7	11	14	15	13	12	14	14
	X	14	12	15	15	15	13	13	13	12	8	9	14	12	7	11	12	13	12	9	11	17

N12																						
0	Y	15	17	17	15	15	14	14	14	14	10	10	14	12	7	14	13	13	13	10	14	18
N12	X	15	12	15	13	14	15	13	13	10	8	9	15.2	13	11	11	14	13	13	9	11	14
1	Y	15	12	16	14	15	17	14	14	11	10	10	17	13	12	13	15	14	13	13	13	17
N12	X	15	12	11	13	14	14	13	14	13	8	9	13	12	7	11	14	13	12	12	12	14
2	Y	15	12	15	15	14	15	13	15	14	10	10	15	12	7	11	14	14	13	13	14	17
N12	X	14	12	11	13	14	13	14	14	11	12	8	15	12	11	11	15	10	12	10	12	18
3	Y	15	17	11	14	15	13	14	15	12	13	8	16.2	13	12	14	15	14	13	11	13	18
N12	X	16	12	15	14	14	12	12	15	10	8	8	13	9	12	11	14	14	12	10	10	17
4	Y	17	15	16	17	16	15	12	16	11	10	9	14.2	9	12	11	16	14	13	12	12	17
N12	X	15	12	11	13	14	15	13	15	10	10	8	14	11	7	11	14	13	12	12	13	14
5	Y	15	12	11	13	15	16	14	15	14	12	9	14.2	13	7	15	15	14	13	13	13	14
N12	X	14	12	11	12	14	16	13	14	11	12	9	13	12	7	11	14	10	12	12	12	14
6	Y	16	16	11	15	15	16	14	15	11	12	9	14	13	12	14	14	13	13	12	13	18
N12	X	14	12	11	13	14	15	13	16	10	10	9	13	11	7	11	10	14	13	12	11	14
7	Y	15	16	11	13	15	15	13	16	11	11	9	14	12	12	14	14	15	13	12	12	14
N12	X	14	12	11	14	14	13	13	13	11	10	8	15.2	12	7	11	14	14	12	11	11	17
8	Y	17	16	11	16	16	14	14	15	11	12	8	16.2	13	7	11	16	16	13	12	12	17
N12	X	14	16	20	13	14	13	14	14	10	8	8	15.2	9	7	11	14	14	10	9	11	17
9	Y	14	17	20	13	15	16	14	14	12	10	11	15.2	12	7	12	16	14	12	13	13	17
N13	X	14	13	11	13	14	12	10	13	10	8	8	13	11	7	12	14	11	13	9	12	17
0	Y	14.3	16	15	14	15	15	13	14	10	8	10	13.2	12	12	15	16	11	15	9	13	17
N13	X	14	16	15	13	13	13	13	14	11	8	8	13	11	12	11	14	10	11	11	12	14
1	Y	14	16	16	14	15	14	14	15	12	12	8	14	14	12	11	14	13	11	12	12	18
N13	X	14	16	15	12	14	15	13	13	10	12	8	14	11	11	11	13	14	11	9	14	14
2	Y	14	17	17	15	14	15	13	14	11	13	8	15	12	13	13	16	15	13	13	14	23
N13	X	17	12	15	13	14	16	10	15	11	12	8	14	12	7	14	13	14	11	11	11	14
3	Y	18	17	16	13	15	17	14	16	12	13	10	14	13	11	14	13	14	13	12	11	14
N13	X	14	12	16	14	15	13	13	13	10	10	8	15	12	11	11	13	14	13	11	11	14
4	Y	14.3	17	17	17	15	16	13	15	12	10	10	15.2	12	11	15	14	14	13	12	12	17
	X	14.3	12	15	13	15	13	10	14	10	10	9	13	12	7	11	13	10	13	12	11	14

N13																						
5	Y	16	12	15	14	15	16	14	15	11	12	9	15.2	13	12	11	14	13	14	12	12	18
N13	X	14	12	11	13	10	11	13	14	10	10	9	15	12	7	11	14	10	10	11	12	17
6	Y	15	17	15	14	14	15	13	15	14	10	10	15.2	12	12	14	14	14	13	12	14	18
N13	X	14	12	11	15	13	10	14	14	11	8	8	14	12	7	11	13	13	12	8	11	18
7	Y	14	16	11	16	14	14	15	14	12	12	10	14	13	7	14	14	13	13	9	12	20
N13	X	14	16	11	13	14	14	13	13	12	10	10	14	13	7	11	14	10	12	11	11	17
8	Y	15	17	17	13	14	15	14	15	14	10	10	14	13	12	14	16	14	13	13	15	20
N13	X	14	16	11	14	13	15	13	13	11	10	9	13	12	7	13	14	14	13	12	12	14
9	Y	14	17	17	16	14	16	14	14	11	10	9	14	12	12	14	16	14	13	12	14	17
N14	X	14	16	16	13	12	14	14	15	10	10	9	15.2	11	7	11	14	14	11	9	12	14
0	Y	18	17	17	14	13	15	14	15	14	12	10	15.2	11	12	15	14	14	14	12	14	18
N14	X	14	17	11	15	15	14	13	14	10	8	8	14.2	12	7	11	13	10	13	9	13	14
1	Y	15	17	17	16	15	15	14	14	11	12	8	14.2	12	11	14	14	14	13	11	14	14
N14	X	15	12	11	13	14	15	13	13	12	12	9	14	11	7	11	14	14	12	12	12	14
2	Y	16	12	15	14	15	17	14	13	14	12	10	14	12	7	11	15	14	13	12	14	18
N14	X	14	16	11	13	11	13	14	15	11	11	8	13.2	12	7	11	13	13	12	11	11	14
3	Y	16	17	16	15	14	15	14	16	11	13	9	14.2	13	12	11	14	14	12	11	12	20
N14	X	15	12	15	13	14	14	13	13	10	10	10	15.2	11	7	12	10	11	13	8	12	17
4	Y	16	16	15	14	15	15	13	15	14	10	11	16.2	12	12	14	15	14	13	11	13	18
N14	X	14	15	11	14	13	14	10	13	10	10	8	14	12	11	11	15	11	12	11	11	17
5	Y	17	17	11	15	15	15	12	15	11	12	10	14	12	12	14	15	14	13	11	12	23
N14	X	14	16	11	13	14	12	13	13	11	10	8	13	9	7	11	14	10	13	12	12	14
6	Y	15	17	16	16	14	16	13	13	12	13	9	14.2	11	11	13	14	13	15	12	12	17
N14	X	14	12	15	14	14	14	10	15	10	12	8	14	10	12	11	14	11	13	10	13	17
7	Y	15	17	17	15	14	15	14	15	11.3	13	10	14.2	12	13	14	14	13	13	11	13	20
N14	X	14	12	11	13	14	14	13	13	11	8	10	14.2	9	7	11	13	11	13	13	11	18
8	Y	17	12	11	14	15	15	14	16	11	10	12	15	12	13	13	14	14	15	14	14	18
N14	X	14	12	11	12	13	14	10	13	11	8	8	14	12	7	13	13	10	12	10	13	14
9	Y	15	17	17	14	15	15	14	14	13	13	9	14.2	12	7	14	15	14	13	13	14	14
	X	16	12	11	14	15	12	13	14	11	11	9	14	12	7	11	12	11	13	11	13	17

N15																						
0	Y	16	16	15	16	15	15	14	15	11	13	12	14.2	13	12	13	14	14	13	11	15	17
N15	X	14	17	11	12	14	14	13	14	8	8	9	14.2	10	7	11	13	11	11	12	10	17
1	Y	17	17	15	14	14	15	14	14	11	10	9	16	12	7	13	14	14	18	14	14	23
N15	X	14	16	14	15	15	16	13	15	11	8	9	13	12	7	11	14	13	11	9	12	14
2	Y	18	17	15	16	15	16	14	15	11	8	9	14.2	12	11	14	15	14	12	13	14	17
N15	X	14	12	16	13	14	13	12	13	10	10	8	13	9	12	11	14	10	11	10	12	17
3	Y	16	12	17	16	15	15	14	13	10	11	10	14.2	10	13	12	14	14	13	12	14	17
N15	X	14	12	15	13	14	13	13	15	11	8	9	14	12	7	11	13	13	10	9	12	14
4	Y	15	17	17	13	14	14	14	16	11	12	12	17	12	7	11	14	14	12	12	13	18
N15	X	16	12	16	15	14	12	13	13	10	8	9	14.2	11	7	11	14	11	12	11	12	14
5	Y	16	17	17	15	14	14	14	14	10	10	9	15.2	11	12	14	14	14	12	12	12	18
N15	X	15	14	11	13	14	12	14	14	10	11	8	13	12	12	11	13	14	13	11	13	14
6	Y	15	16	16	14	14	13	14	16	11	12	10	15.2	13	12	14	15	14	13	11	13	17
N15	X	14	12	11	15	14	12	13	14	12	10	10	13	12	7	10	14	14	12	9	11	17
7	Y	14	18	11	16	15	16	14	15	13	10	10	13	12	13	11	16	14	12	10	13	18
N15	X	15	12	16	14	14	15	13	13	10	8	9	14	11	7	11	14	14	10	12	12	14
8	Y	15	14	16	15	15	15	13	16	11	12	12	14	12	12	12	16	14	14	12	12	14
N15	X	15	16	11	15	15	14	14	13	11	10	9	13	12	7	13	14	14	12	9	11	14
9	Y	15	17	15	15	15	16	14	14	11	12	10	15.2	13	11	14	14	14	13	11	11	17
N16	X	14	14	11	12	14	16	14	13	10	10	8	14	12	7	13	14	13	12	11	11	14
0	Y	14	17	16	15	15	16	14	16	11	10	9	14	12	7	13	15	14	13	12	12	14
N16	X	15	12	15	13	14	13	14	15	10	10	10	14	11	11	11	13	14	12	12	11	14
1	Y	15	14	16	13	15	15	14	16	11.3	12	10	14.2	13	12	13	15	14	13	12	13	18
N16	X	13	12	15	13	14	13	14	14	10	12	9	13	12	11	11	14	13	12	10	10	14
2	Y	15	18	15	14	15	15	14	15	10	12	9	14	12	12	13	16	14	13	12	12	17
N16	X	14	17	15	13	15	13	12	14	10	12	8	14	12	7	13	12	13	11	12	12	14
3	Y	17	17	16	16	15	16	13	16	14	13	9	15.2	14	7	13	14	13	13	12	12	17
N16	X	14	14	11	12	14	14	13	15	11	10	9	12.2	11	7	13	12	10	12	11	12	17
4	Y	15	17	15	15	14	15	14	15	12	12	9	13	13	12	15	14	14	13	12	12	20
	X	14	12	11	13	14	15	12	14	10	10	10	14	10	10	11	13	11	13	9	12	14

N16																						
5	Y	16	17	11	15	15	15	13	15	13	10	11	15	11	12	12	14	13	13	9	15	20
N16	X	15	17	11	13	14	13	12	15	10	10	9	13	12	11	11	13	10	12	10	11	14
6	Y	15	18	15	13	15	16	14	15	11.3	12	10	14.2	12	12	13	16	14	13	12	12	14
N16	X	16	17	11	13	14	12	13	13	10	12	9	13	12	12	13	14	12	13	11	13	14
7	Y	16	18	15	14	15	15	14	13	14	12	12	14.2	13	12	13	14	14	15	11	15	18
N16	X	16	17	11	14	14	14	13	15	10	10	9	13	12	7	11	13	12	11	9	12	17
8	Y	18	18	11	14	15	16	13	16	11	13	10	15.2	12	7	13	13	13	12	11	12	18
N16	X	14	16	15	13	14	13	13	15	11	8	8	14	11	7	11	14	11	13	11	11	17
9	Y	15	17	16	15	15	14	14	17	14	10	9	15.2	12	12	12	15	14	13	13	14	18
N17	X	14	12	17	12	14	15	14	15	10	10	8	13	12	7	11	13	11	12	10	12	17
0	Y	16	17	17	16	14	16	14	16	10	11	9	15	12	7	14	14	13	12	11	12	17
N17	X	15	17	11	13	14	11	12	15	10	10	8	13	9	12	11	14	13	12	9	12	17
1	Y	15	18	11	13	14	14	14	16	10	11	9	14	12	12	14	15	13	12	12	15	17
N17	X	14	14	11	15	14	13	12	13	10	10	8	14	12	12	13	12	14	12	9	11	14
2	Y	15	17	17	15	14	16	12	14	11	12	9	16.2	12	13	14	14	14	12	11	13	18
N17	X	16	12	16	12	15	13	13	15	10	10	9	13	12	7	11	13	10	13	12	11	14
3	Y	17	17	17	13	15	16	13	16	11	12	11	14.2	14	12	14	14	15	14	12	14	18
N17	X	14	16	16	14	14	12	13	13	9.1	10	10	14	11	12	11	12	12	11	11	12	14
4	Y	15	17	16	14	15	14	14	15	11	10	12	14	12	12	11	16	14	12	12	13	17
N17	X	14	12	15	16	10	14	13	15	10	8	9	14.2	12	7	11	14	10	13	9	11	14
5	Y	15	17	17	16	14	16	14	16	11	11	10	14.2	12	7	15	16	14	13	11	12	17
N17	X	15	12	15	13	15	15	13	14	12	10	9	13	12	11	11	13	11	13	12	12	17
6	Y	16	17	17	13	16	15	14	15	14	12	10	14.2	13	11	14	14	11	14	12	12	20
N17	X	14	16	16	13	13	15	13	15	11	8	9	13	13	7	11	14	10	11	11	12	14
7	Y	14	17	17	14	14	15	14	16	11	12	10	16.2	13	12	11	14	10	13	12	13	20
N17	X	16	17	15	13	14	13	13	13	11	8	9	13	9	12	11	13	14	10	9	11	17
8	Y	16	17	15	16	16	14	14	13	12	10	9	16	11	12	15	14	14	13	12	11	18
N17	X	14	16	11	13	13	13	13	14	11	10	9	14	12	7	11	14	11	13	9	12	14
9	Y	15	18	15	13	16	16	14	15	13	13	11	14	13	13	13	15	14	13	12	12	18
	X	14	16	11	13	14	12	14	13	10	12	9	12.2	12	7	11	13	14	12	11	11	14

N18																						
0	Y	15	16	11	15	14	14	14	14	14	12	9	13	13	11	13	14	14	13	13	12	17
N18	X	15	17	15	14	14	15	14	13	10	8	8	14	12	7	11	14	11	12	9	13	17
1	Y	15	17	16	14	14	16	14	14	13	12	9	15.2	13	12	11	15	14	13	9	14	18
N18	X	15	12	11	13	14	13	13	13	10	8	9	14.2	9	12	12	13	14	10	12	11	18
2	Y	15	17	15	13	16	17	14	15	11	13	10	16	13	12	15	13	14	12	12	11	18
N18	X	15	17	16	13	14	15	13	14	10	10	8	15.2	12	7	11	13	13	13	12	12	14
3	Y	16	17	17	14	15	15	14	16	12	12	10	16	13	12	13	14	15	13	12	12	17
N18	X	15	12	11	15	14	15	14	15	10	12	9	14	12	7	14	13	11	12	11	12	14
4	Y	15	17	17	17	14	15	14	15	12	13	12	14.2	13	7	15	15	14	12	14	13	18
N18	X	14	16	11	13	14	14	12	15	10	12	9	13	12	7	12	15	11	13	12	13	17
5	Y	16	17	16	16	15	15	14	16	11	12	12	14	13	12	13	16	11	13	12	13	18
N18	X	15	16	16	13	14	13	10	13	11	8	9	13	11	12	12	14	13	11	9	11	14
6	Y	15	17	17	16	16	16	14	14	12	10	10	13	13	12	15	16	14	13	12	12	14
N18	X	15	12	11	13	14	13	13	14	10	12	8	14.2	13	7	13	13	11	12	11	12	17
7	Y	15	17	16	15	16	15	14	15	12	12	9	16	13	12	13	14	13	13	12	13	17
N18	X	15	17	16	13	13	15	13	14	12	10	10	15.2	12	7	11	14	13	10	10	13	14
8	Y	17	17	17	14	14	16	14	14	12	10	10	15.2	14	7	11	14	14	12	11	14	20
N18	X	14	12	16	13	14	13	14	14	10	8	10	14	12	7	11	15	11	13	9	12	14
9	Y	15	15	17	14	15	16	14	15	14	12	10	14.2	12	12	14	15	14	13	11	13	17
N19	X	15	12	11	14	15	14	14	15	11	8	8	13	12	12	11	13	14	10	10	12	17
0	Y	15	14	17	15	15	14	14	16	12	12	9	14.2	14	12	11	15	15	13	13	12	17
N19	X	14	12	11	15	14	14	14	15	11	10	8	14.2	12	10	11	14	11	13	10	11	14
1	Y	16	17	16	17	16	16	14	16	11	12	11	15.2	12	12	13	14	14	14	11	14	17
N19	X	14	12	11	14	15	15	13	14	12	10	8	13	11	7	14	15	10	12	12	12	17
2	Y	17	12	16	14	15	15	14	14	14	10	8	16.2	12	11	14	16	14	13	13	13	20
N19	X	14	16	16	14	13	13	13	15	10	10	8	14.2	12	7	13	15	14	11	12	12	14
3	Y	15	17	17	15	16	14	14	16	11	12	8	15	13	12	14	15	14	13	12	13	14
N19	X	15	17	11	13	15	13	14	15	11	8	8	14	12	11	11	11	13	10	11	12	17
4	Y	15	17	16	15	15	14	14	17	14	8	8	16	13	12	11	14	14	13	12	13	17
	X	14	12	11	15	14	14	14	15	11	8	9	14.2	12	7	11	13	14	12	10	12	17

N19																						
5	Y	15	12	11	17	16	15	14	15	12	12	12	15	13	12	15	14	14	12	14	13	18
N19	X	14	17	16	12	10	14	14	13	11	8	10	13	12	7	11	13	14	12	13	11	20
6	Y	14	17	17	14	15	16	14	16	12	10	10	13	12	12	11	14	14	13	13	11	23
N19	X	14	12	11	13	15	12	14	14	11	9	11	12	11	12	13	13	13	10	11	11	14
7	Y	16	18	15	15	17	16	14	14	11	13	11	13.2	12	12	14	14	14	11	12	12	14
N19	X	14	17	11	13	15	13	14	13	11	8	10	14	12	11	11	11	11	10	11	12	17
8	Y	15	17	15	15	15	14	14	17	14	13	12	16	12	12	13	14	14	12	12	13	17
N19	X	15	17	17	13	14	14	13	13	10	8	8	13	12	11	11	13	10	12	11	13	17
9	Y	17	18	17	14	14	16	13	15	14	10	10	15	13	11	11	14	14	13	12	13	18
N20	X	15	17	11	14	14	15	13	13	11	12	9	13	12	7	11	10	13	12	10	11	17
0	Y	15	18	16	15	14	16	14	16	14	13	9	13	13	7	13	15	14	12	11	14	20
N20	X	14	14	11	13	14	14	12	13	9.1	10	9	13	10	7	11	12	10	10	9	10	17
1	Y	17	16	11	15	15	16	14	14	14	12	9	15.2	14	11	11	13	14	13	12	14	23
N20	X	14	16	15	13	14	14	12	14	10	8	9	14.2	12	11	11	13	14	13	11.1	11	14
2	Y	17	17	17	14	14	14	14	16	11	14	10	14.2	12	12	13	14	14	13	12	13	17
N20	X	14	12	11	15	14	13	13	13	12	8	10	13	11	7	11	13	13	13	10	11	14
3	Y	15	14	16	15	15	13	14	14	13	8	10	14	12	7	11	14	14	13	11	12	14
N20	X	14	14	16	13	14	12	14	13	11	8	8	13	12	7	11	14	10	10	9	12	17
4	Y	17	17	16	15	14	14	14	15	14	10	10	14	12	7	11	14	13	11	11	15	17
N20	X	14	16	11	13	14	14	13	13	11	10	9	15.2	12	7	11	13	14	12	9	12	18
5	Y	14	18	17	13	16	16	14	15	11	10	9	16.2	13	11	13	15	14	13	12	13	19
N20	X	14	12	11	13	14	15	12	13	11	8	8	13	12	12	13	13	14	13	9	13	17
6	Y	15	17	17	14	14	16	14	15	12	13	9	13	14	12	14	14	14	13	14	14	20
N20	X	14	16	16	13	14	16	13	14	10	8	8	13	12	7	11	13	14	11	11	11	17
7	Y	15	17	17	14	15	16	13	15	12	10	10	15	13	12	14	15	14	11	12	14	18
N20	X	14	16	16	15	14	15	14	15	11	8	9	14	12	7	11	10	14	11	10	14	14
8	Y	15	17	17	16	17	16	14	15	11	10	10	14.2	12	7	13	12	14	12	13	16	17
N20	X	14	14	11	13	14	14	13	14	12	10	8	13	12	7	11	14	10	11	11	11	14
9	Y	16	17	16	14	14	15	13	14	13	13	10	13	13	7	13	14	11	13	13	12	17
	X	14	17	11	14	14	14	13	14	11	8	8	14	12	7	11	14	11	10	9	13	14

N21																						
0	Y	15	17	15	15	15	16	14	15	12	12	9	14.2	12	12	14	14	14	12	10	13	17
N21	X	14	12	11	12	14	14	13	13	11	8	8	13	11	7	11	14	13	12	12	12	14
1	Y	17	14	16	14	15	15	14	14	11	10	11	14	12	11	13	14	14	12	12	12	14
N21	X	14	17	11	13	14	14	14	15	11	8	8	13	12	12	11	13	14	13	11.1	12	18
2	Y	15	17	16	14	15	14	14	16	14	10	9	15.2	14	12	14	13	14	13	12	12	18
N21	X	15	17	11	16	16	13	14	13	10	12	9	15.2	10	7	11	13	14	11	9	13	18
3	Y	16	17	16	17	16	17	14	16	13	13	10	16	12	7	14	14	14	13	9	13	18
N21	X	15	12	15	12	13	15	13	14	11	10	8	14	12	7	11	13	10	13	10	12	14
4	Y	16	17	17	16	15	15	14	16	14	13	11	14.2	12	12	14	14	15	13	12	13	14
N21	X	14	16	16	13	14	14	14	13	11	8	10	13	12	12	13	13	14	13	13	12	17
5	Y	15	17	16	14	15	15	14	16	11	10	11	16.2	13	12	13	14	14	13	14	14	18
N21	X	14	16	11	13	15	15	14	15	10	8	9	13	12	7	14	14	14	12	12	12	17
6	Y	15	17	16	14	16	15	14	15	12	13	10	14	13	11	14	14	14	13	12	13	18
N21	X	14	12	14	15	13	12	13	15	10	8	9	13	11	7	14	12	11	10	10	12	14
7	Y	15	17	17	16	15	15	14	15	10	8	11	14	11	7	15	14	14	13	12	12	17
N21	X	14	17	11	12	13	14	13	13	11	10	8	14.2	12	7	13	14	13	11	12	12	14
8	Y	16	17	16	14	14	17	14	14	14	11	9	15	12	13	14	15	15	13	12	14	20
N21	X	15	17	11	13	14	13	13	14	10	10	9	14	12	7	11	10	12	10	9	10	17
9	Y	16	17	17	15	15	14	14	17	14	12	9	14.2	12	12	13	16	14	12	10	12	17
N22	X	14	12	11	11	14	13	13	14	9.1	10	9	13.2	12	7	11	14	13	12	11	11	17
0	Y	16	17	16	15	15	14	14	15	11.3	13	10	15	12	11	15	15	14	12	11	13	20
N22	X	14	12	11	13	13	12	13	14	11	10	8	13.2	10.1	11	11	12	14	10	9	12	14
1	Y	14	18	11	15	14	16	14	14	12	12	9	15	12	12	11	13	14	13	11	14	17
N22	X	15	17	15	12	14	12	13	13	12	10	10	14	12	7	13	14	13	12	12	12	18
2	Y	16	17	16	13	16	14	14	15	12	11	10	14	13	7	14	14	14	13	13	12	18
N22	X	15	17	11	14	13	15	13	13	11	10	9	14	12	7	11	13	11	12	12	12	14
3	Y	17	17	17	15	14	15	14	14	12	15	11	16.2	12	7	14	14	13	13	12	12	20
N22	X	15	12	14	14	14	14	13	13	10	10	10	13	12	7	10	14	13	13	9	13	18
4	Y	16	17	16	15	14	14	14	13	11	12	12	14	13	7	14	15	13	13	12	14	18
	X	14	17	11	12	14	13	13	14	12	12	10	12	12	7	12	14	14	12	9	10	18

N22																						
5	Y	16	20	11	13	16	16	15	15	14	12	11	13	12	11	15	15	15	12	11	13	18
N22	X	14	12	11	13	14	14	13	13	11	8	8	14	11	7	13	13	10	12	9	13	14
6	Y	16	18	17	15	14	15	13	15	12	11	10	15.2	12	12	13	15	13	13	12	15	18
N22	X	16	12	11	13	14	15	13	14	12	12	9	13	12	11	11	14	14	12	11	12	17
7	Y	16	14	16	13	15	16	14	16	14	14	9	14.2	12	12	14	16	14	13	14	13	17
N22	X	14	17	16	12	15	12	13	14	12	8	9	15	11	7	11	12	13	12	9	12	14
8	Y	15	18	16	13	15	12	14	14	14	10	10	15	12	12	11	13	13	12	10	14	20
N22	X	14	12	15	15	13	14	13	15	10	8	9	13	12	7	11	15	13	12	12	12	17
9	Y	15	17	16	15	14	15	15	16	13	12	10	14	12	12	11	16	15	14	13	12	17
N23	X	14	12	17	13	14	13	13	13	11	8	8	14	11	7	13	14	14	13	12	13	14
0	Y	16	16	17	14	15	16	14	14	11	12	9	14.2	11	12	14	14	17	13	13	14	18
N23	X	15	12	16	12	14	13	12	15	10	12	8	14	12	12	11	14	14	12	12	13	14
1	Y	18	16	17	15	15	14	14	15	12	12	10	14.2	12	12	11	15	14	12	12	14	20
N23	X	14	12	16	13	13	14	12	13	10	10	8	13.2	11	12	11	14	13	12	11	11	18
2	Y	15	17	16	15	13	14	13	13	12	11	12	14.2	14	12	14	14	14	13	11	12	20
N23	X	14	17	11	12	13	13	12	14	10	10	9	15	11	7	11	14	13	12	12	11	14
3	Y	14	18	15	13	14	15	13	16	11	12	9	15.2	14	12	12	15	15	13	12	13	17
N23	X	14	12	15	14	14	13	13	15	11	12	11	13	12	11	11	15	14	13	9	11	14
4	Y	16	17	17	14	14	17	13	16	13	12	12	13	12	12	13	15	14	13	13	14	20
N23	X	15	17	15	13	14	13	14	13	10	10	8	13	11	8	11	14	14	12	9	11	17
5	Y	15	17	17	13	14	15	14	15	10	10	12	14.2	12	12	13	14	14	13	9	12	18
N23	X	14	16	11	13	14	12	13	13	11	11	9	13	11	11	11	13	11	13	10	12	14
6	Y	14	17	11	16	14	15	14	14	11	13	9	14	11	12	11	13	14	13	10	14	20
N23	X	14	12	11	12	14	15	13	15	11	12	10	13	10	12	11	14	13	11	11	12	14
7	Y	16	16	11	13	15	16	14	15	14	12	10	13	12	12	13	14	14	13	12	12	18

Supplementary Table S2. The genotype of 95 Yi individuals living in the Sichuan Yi Autonomous Prefecture, southwest China

Number	Amelogenin	D6S474	D12ATA63	D22S1045	D10S1248	D1S1677	D11S4463	D1S1627	D3S4529	D2S441	D6S1017	D4S2408	D19S433	D17S1301	D1GATA113	D18S853	D20S482	D14S1434	D9S1122	D2S1776	D10S1435	D5S2500
	X	14	16	11	15	14	13	14	13	11	8	8	13	12	7	11	13	11	11	11	10	14
N01	X	17	16	16	20	15	14	14	13	14	13	9	13.2	13	7	15	14	14	12	11	14	18
	X	13	17	15	17	14	13	14	13	10	10	9	13	12	7	11	14	13	12	9	12	18
N02	X	14	17	16	19	14	14	14	15	11.3	12	9	13	13	7	11	15	14	12	9	14	18
	X	14	16	11	15	14	14	11	14	10	10	9	14	13	12	11	15	14	12	12	11	14
N03	X	14	17	16	17	16	14	13	16	14	12	9	16.2	13	13	14	15	15	13	13	13	17
	X	14	17	11	15	13	14	13	14	10	8	10	15.2	7	12	11	14	11	11	11	11	14
N04	X	17	18	17	20	14	14	13	15	11.3	13	10	15.2	14	13	14	14	13	13	11	12	20
	X	14	12	15	17	13	14	14	13	12	10	9	14.2	11	7	11	14	13	12	12	12	14
N05	X	15	18	16	19	15	16	14	13	14	12	10	14.2	12	11	14	15	14	13	12	13	20
	X	16	12	15	17	14	10	11	13	10	10	9	14	11	7	14	13	10	10	13	12	17
N06	X	16	12	16	17	14	14	11	16	10	10	10	14	14	12	14	14	13	12	13	13	23
	X	14	12	15	17	14	15	13	13	11	10	9	15.2	11	7	13	13	11	12	12	12	14
N07	X	14	18	16	20	15	16	14	16	12	13	11	15.2	13	7	14	16	11	13	12	12	20
	X	14	12	11	15	14	13	13	13	10	8	8	14	9	7	10	14	10	11	10	10	14
N08	X	15	17	16	17	14	16	13	15	14	10	8	14	11	12	14	15	13	12	12	12	14
	X	15	16	15	19	14	14	11	14	10	12	10	13	11	7	11	14	12	12	12	11	14
N09	X	15	17	17	19	16	16	11	15	11	12	10	13	12	12	12	14	14	13	12	13	14
	X	14	17	11	14	14	14	11	14	10	12	8	13	12	7	11	13	11	12	10	11	17
N10	X	14	18	16	19	15	14	13	15	10	12	11	13	12	11	15	14	14	13	10	12	17
	X	14	16	11	15	13	13	13	15	10	8	8	13	11	7	11	13	13	11	11	14	14
N11	X	16	17	16	18	15	13	14	16	13	11	9	14	11	7	15	16	13	13	12	14	17
	X	14	17	11	13	14	14	13	14	8	8	9	15	10	7	12	13	13	12	12	11	14
N12	X	16	17	11	14	14	16	13	15	12	10	10	16	10	7	15	14	14	13	15	12	20
	X	14	12	16	19	15	15	11	14	10	12	8	13	12	12	10	14	14	13	12	11	17
N13	X	15	17	17	19	15	16	13	15	13	12	10	13	13	13	14	14	16	14	12	11	17
N14	X	14	12	11	15	14	14	11	13	10	8	8	14	11	7	11	14	14	13	10	12	14

	X	15	17	17	19	16	14	14	14	10	12	9	14	12	12	15	14	14	13	11	12	14
	X	15	12	11	17	14	13	12	13	10	12	10	12	12	8	11	10	10	11	10	12	17
N15	X	16	17	15	20	14	13	13	17	15	13	11	14	12	12	14	15	13	13	11	15	18
	X	14	17	16	17	15	13	13	13	11	10	9	13	12	7	14	15	13	11	9	13	14
N16	X	14	17	17	19	15	15	15	14	14	12	11	13	12	7	14	15	14	12	10	14	18
	X	15	12	11	13	15	12	13	13	10	10	10	13	11	7	11	12	11	12	9	12	17
N17	X	16	17	16	17	15	14	14	17	10	10	11	14.2	12	11	13	14	14	13	12	13	17
	X	16	17	11	13	14	14	13	14	11	10	9	13	11	7	13	10	11	12	11	11	14
N18	X	16	18	15	14	14	14	14	16	14	12	9	16.2	14	12	13	14	13	13	11	13	14
	X	15	17	11	15	14	13	13	13	10	8	8	14	11	12	11	14	11	11	9	12	17
N19	X	17	18	15	17	15	15	16	17	11	10	10	14.2	12	12	14	14	13	13	10	13	17
	X	14	12	11	13	15	15	13	13	11	8	8	14	11	7	11	12	10	13	9	12	14
N20	X	15	17	11	15	15	15	14	15	14	12	10	15	11	11	12	12	13	17	10	14	18
	X	14	15	11	17	14	14	13	17	10	10	8	13	11	7	11	14	13	12	11	11	14
N21	X	20	17	15	19	15	14	13	17	11	12	9	14.2	12	12	13	16	14	13	12	15	14
	X	14	12	11	12	14	15	14	13	11	10	9	13	12	7	13	13	14	12	8	13	14
N22	X	16	17	15	17	15	15	14	13	13	12	10	14	14	11	14	15	14	14	10	14	17
	X	14	16	16	15	15	13	13	13	10	8	9	13	11	12	11	13	13	12	11	12	14
N23	X	14	17	16	17	16	16	14	14	14	10	10	16.2	12	12	14	14	14	13	12	14	18
	X	15	12	11	12	10	16	13	15	10	10	9	13.2	12	12	13	14	10	11	11	12	17
N24	X	17	17	15	17	16	16	13	16	10	10	10	16.2	13	12	14	15	14	12	14	12	20
	X	14	16	11	15	14	14	13	13	10	8	9	13	9	7	11	10	11	13	12	12	14
N25	X	15	17	17	17	14	15	14	16	10	10	10	14.2	11	11	13	16	14	13	12	13	18
	X	17	12	11	14	14	14	13	13	11	10	9	13	9	7	14	15	13	10	10	13	17
N26	X	17	16	19	15	15	15	14	14	12	10	11	13	11	11	14	15	14	12	12	14	20
	X	14	16	11	17	14	13	13	15	10	10	10	14.2	11	11	10	14	11	12	12	12	14
N27	X	14	17	15	17	15	15	15	15	10	10	11	16	12	11	11	14	14	13	12	13	18
	X	14	12	11	13	14	13	12	13	9	10	9	14	9	12	10	10	10	13	10	11	17
N28	X	17	16	15	17	14	15	14	15	14	10	10	15.2	12	13	15	11	14	13	12	11	20
	X	14	12	17	13	14	13	12	13	12	8	9	13	11	11	13	13	14	13	11	11	14
N29	X	17	12	17	15	15	16	13	16	13	10	10	15.2	12	12	13	14	14	13	12	13	17

	X	14	12	11	13	14	13	13	13	11	10	9	15	12	7	12	10	12	12	9	11	17
N30	X	17	16	11	14	15	15	15	15	14	12	11	15.2	13	12	13	13	13	12	13	12	18
	X	14	16	15	15	12	14	13	13	13	8	8	13	11	11	11	12	11	10	10	12	17
N31	X	16	17	17	17	15	14	13	16	14	10	9	14	12	12	11	14	14	13	12	12	18
	X	14	15	17	15	14	14	13	14	11	8	9	14.2	12	7	11	14	10	10	14	11	17
N32	X	16	16	17	19	16	16	14	16	11	12	12	15	13	11	11	14	14	13	14	14	20
	X	15	16	11	15	14	12	12	14	11	8	9	12	12	7	11	12	14	12	11	12	14
N33	X	15	17	17	20	14	13	13	16	11	13	9	15	12	12	11	13	14	13	11	14	17
	X	15	16	11	14	14	14	13	15	12	10	10	13	10	12	11	14	11	11	11	12	14
N34	X	15	18	17	15	14	16	13	16	12	10	10	15	12	12	14	15	14	11	12	13	20
	X	14	12	11	13	15	14	13	14	11	8	8	13	11	7	12	14	11	11	11	12	17
N35	X	15	12	16	13	15	15	14	15	12	12	8	16	12	12	13	15	13	13	12	13	18
	X	14	12	17	13	14	14	12	14	10	8	9	13	12	7	11	12	14	13	12	12	17
N36	X	17	17	17	17	14	14	13	14	10	10	9	14	13	7	13	14	14	13	13	13	18
	X	15	12	11	11	14	15	12	14	11.3	10	9	13	11	7	11	13	13	12	11	11	14
N37	X	15	16	17	15	14	16	14	15	12	12	10	14.2	13	11	11	14	14	12	11	13	18
	X	14	17	15	13	14	14	13	15	11	10	9	15	11	7	13	13	13	11	11	11	17
N38	X	15	17	17	15	14	15	14	15	12	13	10	15.2	12	7	14	15	14	12	12	12	18
	X	14	12	11	11	14	14	15	15	10	8	9	13	10	7	11	11	13	12	11	12	14
N39	X	15	16	11	13	16	14	15	16	15	12	9	14	12	7	11	14	14	13	12	13	17
	X	15	12	16	13	15	16	13	13	12	10	10	14	11	7	13	14	14	13	10	11	14
N40	X	16	16	17	15	15	17	14	15	15	13	11	16	12	12	13	15	14	13	11	14	18
	X	14	12	15	13	14	14	11	13	11.3	8	9	14	11	11	12	13	14	12	10	11	20
N41	Y	14	17	16	15	15	17	13	14	14	12	10	14.2	13	12	13	14	14	12	11	12	20
	X	15	17	11	13	13	14	13	13	10	8	9	14	7	12	11	15	11	10	12	12	14
N42	Y	15	18	15	14	14	14	14	15	10	12	11	15	13	13	14	16	11	15	12	13	14
	X	14	12	15	13	15	13	12	15	10	10	9	9	10	7	11	15	11	12	11	11	14
N43	Y	15	12	17	13	15	13	13	15	11	12	11	13	12	11	12	15	13	13	12	11	17
	X	16	16	11	13	14	14	13	13	11	8	10	13.2	10	7	14	14	14	13	11	11	18
N44	Y	17	17	11	15	16	15	13	16	12	12	10	15.2	10	12	15	15	14	16	12	14	18
N45	X	15	17	11	13	15	14	13	13	10	12	9	13	12	7	11	13	14	12	11	13	14

	Y	16	18	16	13	15	15	14	15	13	12	10	16	13	7	13	15	15	13	13	13	17
	X	15	17	15	13	14	14	13	15	10	11	10	13	12	7	11	14	13	12	12	13	14
N46	Y	16	18	16	14	14	14	14	15	14	12	10	14	13	13	11	15	14	12	13	13	14
	X	14	12	17	13	14	15	12	13	11	10	8	14	11	7	11	13	14	13	12	12	18
N47	Y	15	17	17	14	15	16	15	14	15	10	10	15	12	11	14	14	14	13	12	13	20
	X	14	16	11	13	14	15	13	13	10	10	8	13	11	7	10	14	14	13	10	11	14
N48	Y	15	17	17	15	17	16	13	16	10	13	9	15	12	12	11	14	14	13	11	13	18
	X	14	12	11	13	14	14	10	14	11	10	9	13	9	7	11	13	13	12	11	12	17
N49	Y	15	17	16	13	15	14	12	15	11	11	9	14	10	7	13	13	14	13	12	13	18
	X	14	17	15	13	13	14	13	15	10	10	8	12	11	7	11	13	13	12	9	12	14
N50	Y	17	18	15	14	15	15	15	15	10	13	11	14	11	12	11	14	14	13	12	16	14
	X	14	12	15	13	14	14	13	13	10	10	8	13	12	7	11	14	13	12	11	11	18
N51	Y	17	12	16	13	16	17	13	15	11	12	10	14.2	13	11	12	14	14	12	12	12	20
	X	14	14	14	13	14	13	14	14	11	10	9	12	11	7	11	10	13	13	11	12	17
N52	Y	16	17	16	13	15	13	14	16	13	12	10	14	11	11	14	14	14	13	13	13	18
	X	14	12	15	13	14	13	13	14	11	10	9	14.2	10	11	11	14	13	12	11	12	14
N53	Y	15	12	16	13	15	16	14	15	13	12	10	16.2	12	12	11	15	14	13	13	12	18
	X	14	12	11	12	13	13	13	14	11	8	8	14	13	7	11	14	13	11	11	12	14
N54	Y	15	12	16	13	13	16	14	14	14	12	8	15.2	13	11	13	16	14	12	12	12	17
	X	14	17	11	12	12	13	13	13	12	10	10	13	10	7	11	14	14	11	12	11	14
N55	Y	15	18	16	13	14	13	14	16	14	12	11	15.2	12	12	13	14	14	13	12	13	14
	X	14	14	17	14	15	15	12	13	10	10	10	13	11	12	11	14	11	10	10	11	14
N56	Y	14	18	17	15	15	16	13	15	10	12	10	13.2	12	13	11	15	13	13	11	11	17
	X	16	14	14	13	14	15	13	13	10	10	9	14	12	11	11	13	10	13	12	11	14
N57	Y	18	17	15	14	14	16	13	13	11	12	10	16.2	12	12	14	14	15	13	12	15	18
	X	15	12	11	15	15	14	13	16	11	12	9	13	11	7	13	14	10	13	9	11	17
N58	Y	16	16	17	15	16	16	15	17	14	12	10	15	12	7	14	15	11	14	14	14	17
	X	14	12	11	14	14	15	14	14	10	8	10	13	11	7	12	13	13	13	9	12	14
N59	Y	15	12	16	14	15	15	14	16	10	10	11	14	11	11	13	16	14	17	10	13	18
	X	14	15	11	14	15	9	12	15	11	8	10	13	12	7	12	13	11	11	12	13	16
N60	Y	14	17	17	15	16	15	13	15	12	13	10	13	12	11	15	14	14	12	12	14	18

	X	16	12	16	13	14	13	12	13	10	11	10	14	12	7	11	14	10	10	11	13	14
N61	Y	17	12	16	13	15	13	14	15	14	13	10	15.2	12	11	15	15	14	13	12	14	17
	X	15	16	11	14	14	13	10	15	11.3	10	8	13	12	7	13	13	14	10	12	13	14
N62	Y	17	18	15	14	16	15	14	16	14	10	9	16.2	12	12	14	14	14	13	12	14	17
	X	14	12	11	13	14	13	13	14	10	10	9	14	13	7	11	13	11	12	11	13	18
N63	Y	14	16	16	13	15	14	14	15	14	10	10	14	13	7	12	13	13	13	11	14	20
	X	15	12	11	13	14	15	13	15	11	8	9	13	7	7	11	10	14	10	8	12	14
N64	Y	16	17	17	13	14	16	13	16	12	12	9	14	11	12	13	13	14	12	12	12	17
	X	14	12	15	14	15	13	13	14	11	13	10	13	9	7	11	13	13	10	12	12	17
N65	Y	15	16	15	16	17	15	14	14	11.3	13	10	15.2	10	12	11	14	13	12	12	14	20
	X	15	12	11	14	14	13	13	14	11	11	8	13	10	7	11	13	14	10	9	11	14
N66	Y	17	16	11	16	14	14	14	15	12	12	10	13	12	11	11	14	14	17	10	12	20
	X	14	12	15	13	14	14	12	14	11.3	8	8	13	12	11	11	14	14	12	12	12	14
N67	Y	15	16	16	13	14	15	13	15	14	10	11	14.2	13	12	14	14	14	13	13	13	18
	X	14	12	11	12	14	13	13	13	11	8	8	14	12	7	13	14	13	13	9	13	18
N68	Y	17	14	11	15	14	13	13	13	12	10	12	14	14	12	13	15	13	13	11	14	18
	X	15	12	11	14	13	15	13	13	10	11	10	13	9	11	11	13	11	12	12	12	14
N69	Y	16	17	15	15	14	17	13	16	11	12	10	13	13	11	13	14	14	13	13	13	17
	X	14	17	17	13	14	14	13	14	12	8	8	14.2	11	7	11	14	14	12	9	11	17
N70	Y	15	18	17	15	14	15	15	14	14	10	9	14.2	12	11	13	15	14	17	12	12	17
	X	14	16	11	14	14	13	12	13	11	8	8	13	11	11	11	13	13	11	11	13	14
N71	Y	15	17	15	14	15	15	13	16	11	8	10	14	12	12	11	13	14	12	13	13	18
	X	14	16	11	13	15	14	13	13	11	10	8	14	9	7	14	15	14	12	9	12	14
N72	Y	15	17	16	14	16	16	13	16	14	13	10	14	12	11	15	16	14	13	12	12	17
	X	14	16	11	15	14	15	13	13	11	8	8	14	12	7	11	14	10	13	10	10	14
N73	Y	15	17	16	17	14	16	14	15	13	10	11	16.2	13	7	15	14	12	13	13	12	20
	X	14	17	15	13	14	13	13	13	11	8	9	13	12	11	11	14	11	11	10	12	14
N74	Y	15	18	16	13	15	16	14	15	14	12	10	15	12	11	13	16	13	15	12	14	20
	X	14	12	11	13	14	15	14	16	11	8	10	14.2	11	7	11	14	14	13	10	12	17
N75	Y	14	18	16	13	15	17	14	17	14	8	10	15.2	12	11	12	14	14	13	13	13	17
N76	X	15	12	11	13	14	14	10	14	11	10	9	14	10	7	12	13	14	12	9	12	14

	Y	16	14	17	15	15	15	14	15	11	12	10	15	12	7	14	14	14	13	12	13	18
	X	14	16	16	13	14	13	13	14	11	12	8	14	12	11	11	14	14	12	11	12	14
N77	Y	17	17	17	14	14	14	14	14	12	13	9	14	12	12	11	14	14	13	12	13	17
	X	14	12	11	13	14	15	10	14	10	8	8	13	12	7	14	13	13	10	11	11	18
N78	Y	14	12	16	14	16	16	13	15	11	12	10	14	12	12	15	14	13	13	13	12	18
	X	14	17	17	17	14	13	11	14	11.3	10	10	15	11	7	11	15	14	11	10	11	14
N79	Y	14	18	17	19	14	14	13	15	11.3	12	11	16.2	14	7	14	16	14	13	10	13	18
	X	14	16	11	17	14	13	13	15	10	10	11	14	12	7	11	14	10	11	11	12	14
N80	Y	18	17	16	19	14	15	13	15	11	12	12	16.2	13	11	14	15	14	14	11	13	20
	X	15	12	11	17	15	14	11	13	10	10	10	13	12	7	11	13	14	12	11	12	17
N81	Y	17	16	17	19	16	17	11	14	11.3	12	10	13.2	13	12	13	14	14	12	11	14	17
	X	14	16	15	15	14	13	12	15	11	8	8	13	12	7	11	14	11	12	14	12	17
N82	Y	16	18	17	17	15	14	12	15	11	10	12	13	13	11	14	14	14	13	14	13	18
	X	14	12	11	15	14	14	11	14	11	8	8	15.2	9	7	11	14	11	12	10	12	18
N83	Y	15	16	16	20	15	15	13	16	11	13	10	16.2	10	7	12	15	13	12	14	12	18
	X	15	12	16	17	16	14	13	13	11	10	11	12	12	12	11	13	14	12	12	13	14
N84	Y	15	19	17	19	16	14	14	14	12	12	11	16.2	12	12	14	16	14	13	14	13	17
	X	14	12	11	16	14	13	13	13	11	10	9	13	12	7	11	14	12	13	10	14	14
N85	Y	16	16	16	17	14	14	14	13	14	10	10	16	13	12	14	15	13	13	11	14	20
	X	14	17	15	14	14	12	11	14	10	12	8	14.2	10	7	11	14	14	13	10	13	18
N86	Y	14	18	16	19	14	14	13	16	14	13	9	16.2	12	7	12	14	14	16	12	14	18
	X	15	16	17	15	14	10	11	13	11	8	8	13	12	11	11	14	11	11	10	10	14
N87	Y	17	17	17	19	15	16	13	14	12	12	9	14	13	12	15	14	14	12	11	12	14
	X	14	17	17	19	14	13	12	13	10	12	8	14	12	12	11	15	13	12	10	11	14
N88	Y	17	17	17	19	14	14	13	16	10	12	10	14	13	12	14	16	14	13	10	13	17
	X	14	12	11	17	10	13	11	14	10	10	10	14	11	7	13	15	11	11	14	11	14
N89	Y	17	17	16	17	14	15	14	14	14	10	11	14.2	13	12	14	15	12	13	14	12	14
	X	14	12	11	15	13	12	11	14	11	12	8	15.2	11	7	11	14	14	12	11	14	14
N90	Y	14	16	17	19	15	16	14	15	12	12	10	15.2	12	12	11	14	14	13	11	14	18
	X	14	12	16	15	14	16	13	15	10	8	10	14.2	10	7	14	13	14	11	12	11	17
N91	Y	15	12	17	19	15	16	13	16	14	12	10	14.2	11	12	15	14	14	11	12	14	17

	X	14	17	11	18	14	14	13	13	11	11	8	15.2	9	7	11	15	14	12	12	12	14
N92	Y	14	18	17	19	15	14	13	15	11	12	9	16.2	13	12	14	16	14	13	14	13	14
	X	13	16	17	15	13	13	13	13	9	8	9	13	12	7	15	14	13	12	11	11	17
N93	Y	14	17	17	19	15	15	13	15	10	12	10	13	12	11	16	14	14	13	11	12	18
	X	14	16	15	17	15	13	11	14	11	10	10	13	11	7	11	12	11	13	9	12	17
N94	Y	15	17	17	20	15	15	13	15	14	13	10	15.2	13	12	15	16	13	13	10	13	17
	X	14	12	11	17	15	13	13	16	10	10	10	13	10	7	11	13	13	12	11	11	14
N95	Y	16	12	18	19	15	16	13	16	11	12	11	13	12	7	15	15	13	12	14	13	18

Supplementary Table S3. The p value of the pairwise linkage disequilibrium in all pairs of 21 Non-CODIS STR loci in the Sichuan Tibetan population

	Loci	[01]	[02]	[03]	[04]	[05]	[06]	[07]	[08]	[09]	[10]	[11]	[12]	[13]	[14]	[15]	[16]	[17]	[18]	[19]	[20]	[21]
[01]	D6S474																					
[02]	D12ATA63	0.0182																				
[03]	D22S1045	0.3029	0.0878																			
[04]	D10S1248	0.0226	0.0294	0.1241																		
[05]	D1S1677	0.1019	0.2101	0.2043	0.0034																	
[06]	D11S4463	0.1732	0.1381	0.0365	0.1618	0.0020																
[07]	D1S1627	0.0724	0.0345	0.0321	0.0018	0.0010	0.0910															
[08]	D3S4529	0.6761	0.6056	0.1536	0.0252	0.0025	0.0085	0.2966														
[09]	D2S441	0.4742	0.1301	0.2930	0.0053	0.0245	0.3604	0.0033	0.0288													
[10]	D6S1017	0.1674	0.0523	0.0642	0.1986	0.0321	0.0014	0.1755	0.0405	0.0574												
[11]	D4S2408	0.3619	0.1864	0.0044	0.1712	0.1516	0.1063	0.1807	0.1441	0.0092	0.1174											
[12]	D19S433	0.3447	0.0018	0.0396	0.0082	0.0284	0.0323	0.0593	0.2154	0.6203	0.0223	0.3699										
[13]	D17S1301	0.5119	0.1428	0.0729	0.0391	0.0630	0.0297	0.0027	0.3908	0.2374	0.1376	0.2073	0.0422									
[14]	D1GATA113	0.0066	0.6771	0.1683	0.0102	0.0937	0.1943	0.1915	0.3585	0.0101	0.1204	0.0626	0.5743	0.0806								
[15]	D18S853	0.1169	0.0967	0.1489	0.2014	0.0227	0.3732	0.0198	0.2978	0.1791	0.2364	0.4072	0.8099	0.1045	0.2547							
[16]	D20S482	0.3750	0.6517	0.2692	0.0635	0.0207	0.1020	0.1058	0.0007	0.1416	0.2403	0.1416	0.3505	0.0259	0.1625	0.5313						
[17]	D14S1434	0.0847	0.0805	0.1055	0.0093	0.1259	0.1168	0.0279	0.4287	0.5630	0.4480	0.2000	0.3174	0.3042	0.0812	0.1939	0.6684					
[18]	D9S1122	0.1487	0.0013	0.0067	0.0049	0.0537	0.0325	0.0303	0.1185	0.0361	0.2848	0.2387	0.0141	0.0007	0.2406	0.3485	0.0011	0.0626				
[19]	D2S1776	0.2452	0.1865	0.1141	0.0123	0.0937	0.0014	0.1446	0.0072	0.1847	0.0872	0.4927	0.0302	0.4630	0.3137	0.4683	0.0045	0.0784	0.0055			
[20]	D10S1435	0.1586	0.1642	0.0374	0.1149	0.0493	0.0005	0.0007	0.1454	0.0015	0.0688	0.0768	0.7762	0.0247	0.1785	0.3297	0.0905	0.0733	0.0028	0.0268		
[21]	D5S2500	0.2710	0.0005	0.0957	0.1931	0.0561	0.0000	0.0342	0.3938	0.3510	0.1096	0.0246	0.2296	0.0055	0.6242	0.0465	0.0292	0.4326	0.1735	0.0031	0.0363	

Supplementary Table S4. The p value of the pairwise linkage disequilibrium in all pairs of 21 Non-CODIS STR loci in the Sichuan Yi population

Loci		[01]	[02]	[03]	[04]	[05]	[06]	[07]	[08]	[09]	[10]	[11]	[12]	[13]	[14]	[15]	[16]	[17]	[18]	[19]	[20]	[21]
[01]	D6S474																					
[02]	D12ATA63	0.5443																				
[03]	D22S1045	0.0770	0.0506																			
[04]	D10S1248	0.9365	0.0628	0.0003																		
[05]	D1S1677	0.7881	0.1044	0.9483	0.6329																	
[06]	D11S4463	0.6234	0.3091	0.6992	0.7768	0.3180																
[07]	D1S1627	0.9998	0.9353	0.0359	0.1404	0.9454	0.1479															
[08]	D3S4529	0.2101	0.6436	0.1989	0.8865	0.8336	0.6007	0.0399														
[09]	D2S441	0.1160	0.7116	0.0979	0.4408	0.7493	0.9076	0.1614	0.8885													
[10]	D6S1017	0.5148	0.9908	0.5199	0.0000	0.2280	0.8458	0.8809	0.9507	0.9370												
[11]	D4S2408	0.1853	0.2670	0.8100	0.2380	0.8310	0.1755	0.7805	0.9033	0.9369	0.2176											
[12]	D19S433	0.0873	0.2736	0.2806	0.1285	0.1445	0.7038	0.8491	0.6515	0.8172	0.6962	0.1951										
[13]	D17S1301	0.9418	0.7922	0.6512	0.2876	0.1115	0.7683	0.5219	0.1866	0.5562	0.3045	0.5750	0.4550									
[14]	D1GATA113	0.3110	0.9816	0.7278	0.5932	0.4641	0.1557	0.0439	0.1944	0.2130	0.5615	0.5905	0.2097	0.2072								
[15]	D18S853	0.1901	0.6839	0.7061	0.0557	0.2586	0.2546	0.9898	0.5876	0.2092	0.0944	0.7287	0.9498	0.5639	0.1176							
[16]	D20S482	0.8562	0.6975	0.5188	0.0697	0.1958	0.7053	0.7299	0.9413	0.8689	0.2520	0.1899	0.6925	0.5669	0.4285	0.1417						
[17]	D14S1434	0.2818	0.0857	0.2959	0.4730	0.1869	0.1290	0.8508	0.9299	0.6891	0.9863	0.4468	0.3653	0.6491	0.1592	0.8779	0.6987					
[18]	D9S1122	0.3413	0.8418	0.6645	0.5550	0.5330	0.3140	0.6880	0.7349	0.8535	0.4010	0.3710	0.8769	0.1241	0.2672	0.2310	0.1858	0.1656				
[19]	D2S1776	0.3660	0.7484	0.5178	0.1260	0.1201	0.4858	0.5349	0.7403	0.3779	0.9640	0.2294	0.8046	0.0954	0.8696	0.5586	0.2491	0.3011	0.2758			
[20]	D10S1435	0.1177	0.1241	0.2957	0.0262	0.7046	0.1662	0.4722	0.1993	0.5921	0.0270	0.2590	0.3570	0.6682	0.1122	0.4331	0.5874	0.1333	0.7974	0.6937		
[21]	D5S2500	0.1059	0.3545	0.8468	0.8955	0.3718	0.0462	0.3414	0.5004	0.6684	0.7605	0.4722	0.9355	0.5669	0.5317	0.6934	0.6236	0.3976	0.4200	0.6568	0.4449	

Supplementary Table S5. Allele frequencies of 21 non-CODIS STRs included in AGCU 21+1 amplification kit in Sichuan Tibetan population (n = 237)

Alleles	D6S474	D12ATA63	D22S1045	D10S1248	D1S1677	D11S4463	D1S1627	D3S4529	D2S441	D6S1017	D4S2408	D19S433	D17S1301	D1GATA113	D18S853	D20S482	D14S1434	D9S1122	D2S1776	D10S1435	D5S2500
7									0.0021	0.0021	0.0021			0.4451					0.0042		
8			0.0021	0.0021					0.0042	0.2236	0.2489		0.0021	0.0063				0.0021	0.0084	0.0042	
9			0.0021	0.0021	0.0021	0.0042			0.0042	0.0063	0.3565	0.0021	0.0295			0.0021		0.0021	0.1835	0.0021	
9.1									0.0084												
10			0.0021		0.0211	0.0042	0.0253		0.2637	0.3291	0.2658	0.0021	0.0295	0.0063	0.0084	0.0169	0.0865	0.0696	0.0970	0.0148	
10.1													0.0021								
11		0.0021	0.3249	0.0021	0.0021	0.0042	0.0021		0.3523	0.0527	0.0781		0.1709	0.1667	0.5000	0.0063	0.1055	0.1245	0.2131	0.1962	
11.1																			0.0042		
11.3									0.0127												
12		0.2700		0.0781	0.0042	0.0802	0.0675	0.0021	0.1899	0.2722	0.0485	0.0190	0.5401	0.3439	0.0443	0.0359	0.0148	0.2996	0.3502	0.3629	
12.2												0.0063									
13	0.0042	0.0021		0.3608	0.0759	0.1561	0.3861	0.2342	0.0422	0.1076		0.2574	0.1835	0.0316	0.1962	0.2278	0.2300	0.4451	0.1076	0.2257	0.0021
13.2												0.0359									
14	0.4156	0.0506	0.0169	0.2405	0.5063	0.2595	0.5063	0.2553	0.1118	0.0042		0.2363	0.0380		0.1983	0.4620	0.5169	0.0422	0.0295	0.1582	0.3291
14.2												0.1730									
14.3	0.0063																				
15	0.3418	0.0253	0.1751	0.1941	0.2911	0.2827	0.0127	0.3502	0.0084	0.0021		0.0949	0.0042		0.0506	0.1646	0.0401	0.0105	0.0021	0.0274	
15.2												0.0970									
16	0.1392	0.1498	0.2722	0.0970	0.0907	0.1793		0.1350				0.0253			0.0021	0.0844	0.0042	0.0021		0.0084	0.0021
16.2												0.0380									
17	0.0759	0.4283	0.1941	0.0232	0.0063	0.0274		0.0232				0.0063					0.0021				0.3502
17.2												0.0042									
18	0.0169	0.0696	0.0063			0.0021						0.0021						0.0021			0.2004
19																					0.0042
20		0.0021	0.0042																		0.0823
21																					0.0042
23																					0.0253

Supplementary Table S6. Allele frequencies of 21 non-CODIS STRs included in the AGCU 21+1 kit in the Sichuan Yi Population (n = 95)

Allele	D6S474	D12ATA63	D22S1045	D10S1248	D1S1677	D11S4463	D1S1627	D3S4529	D2S441	D6S1017	D4S2408	D19S433	D17S1301	D1GATA113	D18S853	D20S482	D14S1434	D9S1122	D2S1776	D10S1435	D5S2500
7													0.0158	0.4684							
8									0.0053	0.2053	0.1895			0.0053					0.0105		
9						0.0053			0.0105		0.3000	0.0053	0.0526						0.0842		
10					0.0105	0.0105	0.0211		0.2947	0.3579	0.3737		0.0895		0.0263	0.0368	0.0632	0.0632	0.1579	0.0211	
11			0.3316	0.0105			0.0947		0.3000	0.0368	0.1158		0.2316	0.2053	0.4263	0.0105	0.1421	0.1211	0.2684	0.1947	
11.3									0.0526												
12		0.3158		0.0263	0.0105	0.0211	0.0895		0.1158	0.3053	0.0211	0.0263	0.4105	0.2842	0.0789	0.0368	0.0263	0.3316	0.3316	0.3474	
13	0.0105			0.2737	0.0526	0.2263	0.4895	0.2684	0.0474	0.0947		0.3158	0.1684	0.0368	0.1684	0.1947	0.2474	0.4211	0.0789	0.2684	
13.2												0.0263									
14	0.4421	0.0263	0.0105	0.1368	0.5053	0.3211	0.2526	0.2421	0.1526			0.2421	0.0316		0.2053	0.4474	0.5000	0.0211	0.0632	0.1474	0.3684
14.2												0.1053									
15	0.2842	0.0158	0.1789	0.1947	0.3211	0.2211	0.0474	0.2895	0.0211			0.0737			0.0895	0.2000	0.0158	0.0105	0.0053	0.0158	
15.2												0.1000									
16	0.1316	0.2053	0.2263	0.0158	0.0895	0.1632	0.0053	0.1632				0.0316			0.0053	0.0737	0.0053	0.0105		0.0053	0.0053
16.2												0.0737									
17	0.1158	0.3211	0.2421	0.1684	0.0105	0.0316		0.0368										0.0211			0.2842
18	0.0105	0.1105	0.0053	0.0105																	0.2316
19		0.0053	0.0053	0.1263																	
20	0.0053			0.0368																	0.1053
23																					0.0053

Supplementary Table S7. The Fst and corresponding p value between the Sichuan Tibetan and 27 Chinese reference populations

Loci	Fst and p	LST	QHS	HBTJ	YNB	NXH	AU	ILK	FJS	IMM	NH	GNH	YNY	HNL	GGH
D1GATA113	Fst	-0.0028	-0.0060	0.0060	-0.0018	-0.0027	-0.0016	-0.0011	0.0005	-0.0005	0.0048	0.0033	0.0105	0.0030	-0.0004
	p	0.5646	0.9831	0.1439	0.4767	0.7142	0.6416	0.4361	0.3256	0.4275	0.1069	0.1465	0.0646	0.1308	0.4182
D1S1627	Fst	0.0543	0.0444	0.0768	0.0607	0.0678	0.0428	0.0256	0.1409	0.0522	0.0617	0.0577	0.0641	0.0893	0.0866
	p	0.0006	0.0006	0.0000	0.0002	0.0000	0.0000	0.0103	0.0000	0.0000	0.0000	0.0000	0.0000	0.0000	0.0000
D1S1677	Fst	-0.0017	0.0042	0.0005	0.0109	-0.0011	0.0033	0.0056	0.0000	0.0103	0.0016	-0.0004	-0.0042	0.0090	-0.0008
	p	0.4917	0.1678	0.3215	0.0612	0.4742	0.1014	0.1266	0.3642	0.0095	0.2428	0.4016	0.7764	0.0108	0.5048
D2S441	Fst	0.0014	-0.0001	0.0068	0.0000	-0.0015	0.0023	0.0012	-0.0041	-0.0006	-0.0017	-0.0024	0.0048	0.0095	0.0052
	p	0.2993	0.3858	0.0938	0.3907	0.5867	0.1311	0.2965	0.9196	0.4866	0.6495	0.8024	0.1326	0.0049	0.0267
D2S1776	Fst	-0.0041	0.0001	0.0010	0.0045	0.0023	0.0036	0.0079	0.0011	0.0056	0.0010	0.0031	0.0135	0.0126	0.0081
	p	0.8183	0.3888	0.3145	0.1526	0.1859	0.0725	0.0632	0.2957	0.0277	0.2882	0.1263	0.0220	0.0015	0.0078
D3S4529	Fst	0.0067	-0.0028	-0.0051	-0.0021	0.0002	0.0061	-0.0028	0.0141	-0.0003	0.0001	0.0026	-0.0024	0.0071	-0.0008
	p	0.1136	0.6498	0.8668	0.5611	0.3658	0.0282	0.6481	0.0119	0.4266	0.3714	0.1687	0.5967	0.0158	0.5159
D4S2408	Fst	0.0015	0.0159	-0.0036	-0.0063	0.0003	0.0145	0.0049	-0.0014	0.1214	0.0049	0.0035	0.0218	0.0220	0.0162
	p	0.2974	0.0148	0.7173	0.9836	0.3460	0.0010	0.1462	0.5329	0.0000	0.0888	0.1289	0.0048	0.0000	0.0006
D5S2500	Fst	-0.0051	0.0005	0.0005	-0.0024	0.0047	0.0067	-0.0012	0.0051	-0.0010	0.0016	0.0045	-0.0031	0.0182	0.0058
	p	0.8671	0.3377	0.3457	0.5674	0.1117	0.0253	0.4704	0.1162	0.5601	0.2338	0.0934	0.6566	0.0004	0.0392
D6S474	Fst	-0.0049	-0.0029	0.0090	-0.0040	0.0014	0.0051	0.0032	0.0014	-0.0006	0.0021	-0.0021	0.0000	0.0075	0.0059
	p	0.8332	0.6511	0.0724	0.7214	0.2587	0.0470	0.2048	0.2708	0.4691	0.2184	0.7018	0.3769	0.0186	0.0383
D6S1017	Fst	0.0037	-0.0051	-0.0024	0.0070	0.0018	0.0092	-0.0003	0.0113	-0.0009	0.0015	-0.0027	0.0009	0.0205	0.0145
	p	0.1906	0.9444	0.6019	0.0996	0.2213	0.0072	0.4113	0.0233	0.5520	0.2492	0.8554	0.3236	0.0000	0.0009
D9S1122	Fst	-0.0057	-0.0029	-0.0026	-0.0058	0.0117	0.0068	0.0054	-0.0032	0.0024	0.0015	0.0044	0.0141	0.0037	0.0018
	p	0.9516	0.6531	0.6034	0.9640	0.0164	0.0242	0.1285	0.7735	0.1412	0.2363	0.0855	0.0264	0.0893	0.1721
D10S1248	Fst	-0.0030	-0.0031	-0.0052	-0.0044	-0.0035	0.0022	0.0000	0.0210	-0.0019	-0.0024	-0.0022	-0.0040	-0.0027	-0.0017
	p	0.6809	0.7188	0.9197	0.8436	0.9266	0.1439	0.3931	0.0018	0.8262	0.7595	0.7795	0.7999	0.9864	0.7566
D10S1435	Fst	-0.0012	-0.0012	-0.0023	-0.0042	0.0012	0.0006	-0.0034	-0.0011	-0.0009	0.0023	-0.0003	0.0062	0.0005	0.0004
	p	0.4851	0.4952	0.6039	0.8073	0.2703	0.3071	0.7473	0.5035	0.5771	0.1812	0.4219	0.1110	0.3168	0.3270
D11S4463	Fst	-0.0019	0.0112	0.0001	0.0073	0.0035	0.0130	0.0165	0.0253	0.0034	0.0014	0.0052	0.0166	0.0097	0.0118
	p	0.5692	0.0292	0.3930	0.0797	0.1240	0.0005	0.0076	0.0004	0.0760	0.2511	0.0610	0.0082	0.0047	0.0011

D12ATA63	Fst	0.0026	-0.0040	0.0021	-0.0017	0.0039	0.0129	0.0047	0.0159	0.0050	0.0077	0.0033	0.0087	0.0610	0.0334
	p	0.2387	0.8268	0.2470	0.5324	0.1167	0.0013	0.1454	0.0084	0.0459	0.0372	0.1267	0.0593	0.0000	0.0000
D14S1434	Fst	0.0045	0.0143	-0.0030	0.0119	0.0136	0.0117	0.0187	-0.0040	0.0111	0.0127	0.0182	-0.0028	0.0129	0.0009
	p	0.1692	0.0216	0.6297	0.0418	0.0118	0.0033	0.0088	0.8965	0.0050	0.0084	0.0016	0.6308	0.0026	0.2579
D17S1301	Fst	0.0007	-0.0032	0.0043	-0.0028	-0.0006	0.0044	0.0224	0.0012	0.0085	0.0068	0.0022	0.0287	-0.0018	0.0038
	p	0.3256	0.7038	0.1666	0.6048	0.4406	0.0602	0.0043	0.2724	0.0114	0.0488	0.1835	0.0029	0.7639	0.0719
D18S853	Fst	-0.0018	0.0067	0.0006	0.0263	0.0033	0.0032	0.0070	0.0145	0.0122	0.0106	0.0052	0.0118	0.0002	0.0032
	p	0.5115	0.1023	0.3352	0.0034	0.1527	0.0994	0.1021	0.0157	0.0029	0.0170	0.0737	0.0398	0.3406	0.0994
D19S433	Fst	0.0030	-0.0011	-0.0023	-0.0002	-0.0024	0.0057	-0.0015	0.0104	0.0023	0.0017	0.0017	0.0044	0.0021	0.0011
	p	0.1916	0.5442	0.6736	0.4284	0.8363	0.0123	0.5860	0.0123	0.1104	0.2030	0.1956	0.1265	0.1159	0.2261
D20S482	Fst	-0.0048	-0.0018	-0.0041	-0.0018	0.0001	0.0021	-0.0002	0.0048	0.0021	0.0017	0.0007	0.0107	0.0124	0.0074
	p	0.8684	0.5490	0.7937	0.5242	0.3615	0.1544	0.4057	0.1208	0.1495	0.2218	0.3020	0.0445	0.0025	0.0212
D22S1045	Fst	-0.0026	0.0660	0.0196	0.0007	0.0064	0.0210	0.0015	0.0980	0.0060	0.0399	0.0096	0.0868	0.0386	0.0213
	p	0.6099	0.0000	0.0058	0.3372	0.0578	0.0000	0.2889	0.0000	0.0212	0.0000	0.0122	0.0000	0.0000	0.0000

LST: Lhasa-Tibetan; QHS: Qinghai-Salar; HBTJ: Hubei-Tujia; YNB: Yunnan-Bai; NXH: Ningxia-Han; AU: Kuqa-Uyghur; ILK: Ili-Kazakh; FJS: Fujian-She; IMM: Inner-Mongolia-Mongolian; NH: Northern-Han; GNH: Guanzhong-Han; YNY: Yunnan-Yi; HNL: Hainan-Li; GGH: Guangdong-Han; HNH: Hunan-Han; HDH: Huadong-Han; BJH: Beijing-Han; ZJH: Zhejiang-Han; IMR: Inner-Mongolia-Russian; SDH: Shandong-Han; LNH: Liaoning-Han; HNH-2: Henan-Han-2; GSYG: Gansu-Yugu; XJXO: Xinjiang-Xibe; HNH-1: Henan-Han-1; SCH: Sichuan-Han; SCY: Sichuan Yi

Supplementary Table S7. (Continue) The Fst and corresponding p value between the Sichuan Tibetan and 27 Chinese reference populations

Loci	Fst and p	HNH	HDH	BJH	ZJH	IMR	SDH	LNH	HNH-2	GSYG	XJXO	HNH-1	SCH	SCY
D1GATA113	Fst	0.0022	-0.0010	0.0049	0.0031	-0.0054	0.0034	0.0014	0.0023	-0.0043	0.0047	-0.0018	-0.0013	-0.0024
	p	0.1683	0.4699	0.0708	0.1239	0.9097	0.0907	0.2504	0.1368	0.9368	0.0544	0.5752	0.5620	0.5216
D1S1627	Fst	0.0754	0.0974	0.0607	0.0486	0.0625	0.0609	0.0497	0.0567	0.0871	0.0517	0.0280	0.0590	0.0569
	p	0.0000	0.0000	0.0000	0.0000	0.0001	0.0000	0.0000	0.0000	0.0000	0.0000	0.0019	0.0000	0.0004
D1S1677	Fst	-0.0006	-0.0010	0.0027	-0.0006	-0.0043	0.0008	0.0006	0.0004	0.0145	0.0010	-0.0012	0.0021	-0.0057
	p	0.4581	0.4915	0.1354	0.4671	0.8165	0.2475	0.3077	0.2966	0.0102	0.2313	0.5096	0.1709	0.8913
D2S441	Fst	0.0018	0.0012	-0.0019	-0.0017	0.0001	-0.0010	-0.0033	-0.0013	-0.0012	-0.0022	0.0025	-0.0008	0.0008
	p	0.1644	0.2632	0.8130	0.7664	0.3811	0.6373	0.9034	0.7259	0.5211	0.9627	0.1824	0.5281	0.3373
D2S1776	Fst	0.0010	0.0007	0.0026	0.0082	0.0025	0.0029	0.0019	0.0029	0.0138	0.0064	0.0108	0.0064	0.0056
	p	0.2528	0.3170	0.1188	0.0083	0.2281	0.0782	0.2175	0.0768	0.0068	0.0125	0.0118	0.0257	0.1359
D3S4529	Fst	-0.0027	0.0005	-0.0008	-0.0016	-0.0009	0.0010	-0.0015	0.0012	0.0121	0.0003	-0.0013	-0.0021	-0.0023
	p	0.9611	0.3472	0.5269	0.7007	0.4657	0.2260	0.5616	0.2145	0.0163	0.3346	0.5591	0.7821	0.5657
D4S2408	Fst	0.0046	0.0080	0.0035	0.0044	0.0106	0.0036	-0.0032	0.0015	0.0115	0.0013	0.0110	0.0059	0.0066
	p	0.0523	0.0351	0.0890	0.0638	0.0469	0.0648	0.8454	0.1929	0.0194	0.2158	0.0135	0.0395	0.1298
D5S2500	Fst	0.0060	0.0060	0.0068	0.0021	0.0114	0.0024	0.0030	0.0063	0.0015	0.0057	0.0022	0.0059	-0.0021
	p	0.0387	0.0681	0.0299	0.1625	0.0429	0.1294	0.1648	0.0230	0.2628	0.0273	0.2023	0.0492	0.5376
D6S474	Fst	0.0089	0.0007	0.0026	-0.0008	0.0013	0.0022	-0.0031	0.0033	0.0085	0.0016	0.0001	0.0036	-0.0030
	p	0.0126	0.3083	0.1325	0.5178	0.2941	0.1299	0.8134	0.0768	0.0597	0.1777	0.3633	0.1126	0.5936
D6S1017	Fst	0.0073	0.0040	0.0007	0.0058	0.0005	0.0016	0.0022	0.0021	-0.0016	0.0026	-0.0006	0.0026	-0.0055
	p	0.0160	0.1157	0.2838	0.0338	0.3551	0.1736	0.2009	0.1360	0.5639	0.1109	0.4571	0.1439	0.8937
D9S1122	Fst	0.0014	-0.0004	0.0011	0.0010	0.0038	0.0014	-0.0023	0.0026	0.0037	0.0050	0.0005	0.0011	-0.0058
	p	0.2141	0.4206	0.2407	0.2550	0.1738	0.1833	0.6813	0.0991	0.1535	0.0348	0.3283	0.2472	0.9274
D10S1248	Fst	-0.0024	-0.0035	-0.0003	-0.0009	-0.0052	-0.0010	-0.0033	-0.0007	0.0002	-0.0018	-0.0023	-0.0028	0.0335
	p	0.9414	0.9486	0.4202	0.5538	0.9453	0.6247	0.8909	0.5440	0.3667	0.8690	0.7541	0.9529	0.0000
D10S1435	Fst	-0.0011	-0.0017	-0.0002	0.0187	0.0019	-0.0001	-0.0014	0.0010	0.0042	0.0002	0.0037	-0.0013	-0.0057
	p	0.6015	0.6346	0.4114	0.0000	0.2636	0.4108	0.5772	0.2206	0.1195	0.3526	0.1230	0.6220	0.9191
D11S4463	Fst	0.0096	0.0052	0.0065	0.0039	0.0067	0.0051	0.0033	0.0072	0.0214	0.0049	0.0075	0.0016	0.0026
	p	0.0041	0.0727	0.0172	0.0645	0.0850	0.0209	0.1338	0.0064	0.0006	0.0269	0.0266	0.2115	0.2327

D12ATA63	Fst	0.0209	0.0121	0.0043	0.0159	-0.0040	0.0085	0.0019	0.0103	0.0344	0.0052	0.0056	0.0133	0.0056
	p	0.0000	0.0083	0.0658	0.0005	0.8199	0.0066	0.2219	0.0028	0.0001	0.0321	0.0732	0.0025	0.1377
D14S1434	Fst	0.0076	0.0022	0.0121	0.0049	0.0139	0.0060	0.0066	0.0077	-0.0006	0.0056	0.0077	0.0051	-0.0059
	p	0.0176	0.1938	0.0032	0.0551	0.0220	0.0204	0.0615	0.0104	0.4375	0.0284	0.0438	0.0560	0.9250
D17S1301	Fst	0.0047	0.0040	0.0045	0.0035	0.0031	0.0027	0.0078	0.0070	-0.0033	0.0026	0.0281	0.0044	0.0100
	p	0.0504	0.1152	0.0651	0.0849	0.1913	0.0984	0.0442	0.0132	0.8298	0.1059	0.0001	0.0740	0.0639
D18S853	Fst	0.0022	0.0038	0.0048	0.0058	0.0022	0.0063	0.0067	0.0030	-0.0008	0.0066	0.0225	0.0042	-0.0009
	p	0.1451	0.1301	0.0561	0.0430	0.2399	0.0168	0.0598	0.0857	0.4557	0.0171	0.0009	0.0830	0.4418
D19S433	Fst	0.0031	0.0016	0.0059	0.0019	0.0014	0.0029	0.0028	0.0031	0.0069	0.0024	0.0037	0.0028	-0.0009
	p	0.0650	0.2057	0.0173	0.1392	0.2785	0.0620	0.1438	0.0445	0.0274	0.0821	0.0892	0.0951	0.4946
D20S482	Fst	0.0011	-0.0005	0.0042	0.0032	0.0033	0.0004	0.0142	0.0017	0.0062	0.0009	0.0022	0.0087	-0.0053
	p	0.2451	0.4399	0.0691	0.1097	0.1951	0.3079	0.0078	0.1649	0.0708	0.2491	0.1956	0.0152	0.8678
D22S1045	Fst	0.0183	0.0149	0.0085	0.3024	0.0866	0.0107	0.0066	0.0080	0.0019	0.0058	0.0040	0.0179	-0.0039
	p	0.0000	0.0017	0.0091	0.0000	0.0000	0.0005	0.0535	0.0038	0.2495	0.0180	0.1196	0.0003	0.7189

LST: Lhasa-Tibetan; QHS: Qinghai-Salar; HBTJ: Hubei-Tujia; YNB: Yunnan-Bai; NXH: Ningxia-Han; AU: Kuqa-Uyghur; ILK: Ili-Kazakh; FJS: Fujian-She; IMM: Inner-Mongolia-Mongolian; NH: Northern-Han; GNH: Guanzhong-Han; YNY: Yunnan-Yi; HNL: Hainan-Li; GGH: Guangdong-Han; HNH: Hunan-Han; HDH: Huadong-Han; BJH: Beijing-Han; ZJH: Zhejiang-Han; IMR: Inner-Mongolia-Russian; SDH: Shandong-Han; LNH: Liaoning-Han; HNH-2: Henan-Han-2; GSYG: Gansu-Yugu; XJXO: Xinjiang-Xibe; HNH-1: Henan-Han-1; SCH: Sichuan-Han; SCY: Sichuan Yi

Supplementary Table S8. The Fst and corresponding p value between the Sichuan Yi and 27 Chinese reference populations

Loci	Fst and p	LST	QHS	HBTJ	YNB	NXH	AU	ILK	FJS	IMM	NH	GNH	YNY	HNL	GGH
D1GATA113	Fst	-0.0092	-0.0035	-0.0019	0.0006	-0.0061	0.0054	0.0101	0.0103	-0.0010	-0.0020	-0.0045	0.0226	-0.0007	0.0000
	p	0.9747	0.5513	0.4535	0.3336	0.8711	0.1414	0.1088	0.0994	0.4261	0.4996	0.7282	0.0330	0.4118	0.3622
D1S1627	Fst	0.0108	-0.0029	0.0043	0.0074	0.0053	0.0005	0.0063	0.0313	0.0052	0.0052	0.0047	0.0015	0.0212	0.0097
	p	0.1039	0.5336	0.2031	0.1446	0.1592	0.3304	0.1641	0.0068	0.1435	0.1576	0.1613	0.2990	0.0066	0.0604
D1S1677	Fst	-0.0035	0.0081	-0.0039	0.0056	-0.0061	0.0022	0.0050	-0.0018	0.0048	0.0016	-0.0035	-0.0066	0.0107	-0.0039
	p	0.5490	0.1346	0.5808	0.1919	0.8912	0.2414	0.1800	0.4617	0.1428	0.2804	0.6392	0.7875	0.0455	0.7586
D2S441	Fst	-0.0052	-0.0033	-0.0042	0.0005	0.0060	-0.0036	-0.0028	0.0044	0.0032	-0.0043	0.0049	-0.0005	0.0134	0.0055
	p	0.7523	0.6127	0.6837	0.3621	0.1237	0.8227	0.5663	0.1855	0.1828	0.8084	0.1479	0.4275	0.0109	0.1001
D2S1776	Fst	0.0027	0.0001	0.0042	0.0061	-0.0016	0.0005	-0.0037	0.0062	0.0032	0.0085	0.0030	0.0114	0.0126	0.0108
	p	0.2587	0.3840	0.2012	0.1545	0.5204	0.3572	0.6534	0.1365	0.1835	0.0769	0.2016	0.0796	0.0284	0.0384
D3S4529	Fst	-0.0048	-0.0056	-0.0060	-0.0027	0.0031	-0.0044	-0.0079	0.0069	0.0046	0.0082	0.0108	-0.0053	-0.0039	-0.0046
	p	0.6929	0.7710	0.7605	0.5452	0.2267	0.8649	0.9320	0.1422	0.1436	0.0968	0.0519	0.7354	0.8084	0.8784
D4S2408	Fst	-0.0082	0.0006	0.0006	0.0027	-0.0009	-0.0016	0.0224	0.0053	0.1191	0.0061	-0.0004	-0.0040	-0.0019	-0.0046
	p	0.9157	0.3602	0.3597	0.2761	0.4508	0.5342	0.0171	0.1754	0.0000	0.1370	0.4065	0.6284	0.5681	0.8696
D5S2500	Fst	-0.0067	0.0006	-0.0055	-0.0066	-0.0039	-0.0036	0.0100	0.0039	-0.0044	-0.0058	-0.0035	-0.0042	0.0004	-0.0051
	p	0.8202	0.3507	0.7207	0.7970	0.6753	0.7471	0.1054	0.2146	0.8368	0.8874	0.6609	0.6231	0.3523	0.9217
D6S474	Fst	-0.0012	-0.0065	0.0177	-0.0039	0.0003	0.0079	0.0055	0.0021	0.0022	0.0039	0.0017	-0.0014	0.0148	0.0164
	p	0.4316	0.8488	0.0409	0.5967	0.3673	0.0746	0.1872	0.2702	0.2449	0.2037	0.2727	0.4548	0.0190	0.0172
D6S1017	Fst	0.0033	-0.0072	-0.0047	0.0009	-0.0059	0.0027	-0.0048	0.0040	-0.0051	-0.0026	-0.0065	-0.0051	0.0130	0.0071
	p	0.2493	0.9063	0.6837	0.3466	0.8921	0.2220	0.6789	0.2031	0.9289	0.5661	0.9859	0.7084	0.0264	0.0934
D9S1122	Fst	-0.0082	-0.0078	-0.0049	-0.0085	0.0044	0.0019	0.0041	-0.0034	-0.0012	-0.0022	0.0000	0.0064	-0.0006	-0.0030
	p	0.9464	0.9543	0.6826	0.9626	0.1875	0.2538	0.2237	0.6083	0.4877	0.5481	0.3813	0.1640	0.4194	0.6585
D10S1248	Fst	0.0356	0.0352	0.0322	0.0315	0.0344	0.0308	0.0295	0.0724	0.0348	0.0369	0.0369	0.0360	0.0313	0.0352
	p	0.0005	0.0011	0.0016	0.0025	0.0003	0.0003	0.0023	0.0000	0.0002	0.0006	0.0001	0.0009	0.0002	0.0000
D10S1435	Fst	-0.0048	-0.0055	-0.0062	-0.0092	-0.0028	-0.0042	-0.0059	-0.0070	-0.0034	-0.0023	-0.0058	0.0114	-0.0015	-0.0026
	p	0.6855	0.7770	0.8297	0.9963	0.6148	0.8524	0.8207	0.9597	0.7786	0.5936	0.9524	0.0761	0.5388	0.6627
D11S4463	Fst	0.0038	-0.0013	-0.0007	-0.0017	-0.0031	-0.0014	0.0041	0.0079	-0.0033	-0.0027	-0.0002	0.0036	-0.0024	-0.0022

	p	0.2351	0.4584	0.4253	0.4782	0.6386	0.5178	0.2151	0.1120	0.7481	0.6244	0.4004	0.2428	0.6440	0.6149
D12ATA63	Fst	0.0316	-0.0033	-0.0086	-0.0066	-0.0034	-0.0021	0.0166	-0.0033	-0.0029	-0.0044	-0.0035	-0.0041	0.0190	0.0021
	p	0.0077	0.5920	0.9769	0.8327	0.6606	0.6034	0.0336	0.6098	0.6794	0.7653	0.6918	0.6659	0.0033	0.2387
D14S1434	Fst	0.0015	0.0047	-0.0091	0.0050	0.0044	0.0053	0.0110	-0.0053	0.0040	0.0064	0.0106	-0.0082	0.0053	-0.0052
	p	0.3063	0.1994	0.9862	0.1944	0.1833	0.1252	0.0871	0.7846	0.1723	0.1262	0.0566	0.9616	0.1293	0.9507
D17S1301	Fst	-0.0043	0.0038	-0.0075	0.0053	0.0035	-0.0024	-0.0021	-0.0014	-0.0027	-0.0030	-0.0007	-0.0021	0.0052	-0.0001
	p	0.6456	0.2157	0.9241	0.1898	0.2046	0.6093	0.4993	0.4750	0.6579	0.6451	0.4272	0.5072	0.1338	0.3819
D18S853	Fst	-0.0017	-0.0015	-0.0081	0.0051	-0.0051	-0.0026	-0.0053	0.0090	0.0002	-0.0012	-0.0019	-0.0014	0.0047	-0.0026
	p	0.4763	0.4581	0.9380	0.1973	0.8187	0.6431	0.7452	0.0941	0.3662	0.4649	0.5298	0.4669	0.1433	0.6409
D19S433	Fst	0.0022	-0.0039	-0.0009	-0.0059	-0.0045	0.0002	0.0009	0.0089	-0.0012	-0.0038	-0.0029	-0.0028	-0.0008	-0.0030
	p	0.2758	0.7292	0.4587	0.8773	0.8619	0.3796	0.3532	0.0703	0.5435	0.8040	0.7145	0.6211	0.4925	0.7923
D20S482	Fst	-0.0044	-0.0020	-0.0015	-0.0082	0.0015	0.0031	-0.0007	-0.0002	0.0003	0.0013	-0.0019	0.0140	0.0134	0.0106
	p	0.6478	0.4989	0.4541	0.9390	0.2933	0.1942	0.4173	0.3941	0.3575	0.2950	0.5477	0.0565	0.0235	0.0504
D22S1045	Fst	0.0011	0.0617	0.0211	-0.0036	0.0060	0.0181	0.0081	0.0971	0.0057	0.0370	0.0097	0.0936	0.0315	0.0180
	p	0.3420	0.0000	0.0194	0.6133	0.1317	0.0044	0.1280	0.0000	0.1110	0.0000	0.0575	0.0000	0.0003	0.0059

LST: Lhasa-Tibetan; QHS: Qinghai-Salar; HBTJ: Hubei-Tujia; YNB: Yunnan-Bai; NXH: Ningxia-Han; AU: Kuqa-Uyghur; ILK: Ili-Kazakh; FJS: Fujian-She; IMM: Inner-Mongolia-Mongolian; NH: Northern-Han; GNH: Guanzhong-Han; YNY: Yunnan-Yi; HNL: Hainan-Li; GGH: Guangdong-Han; HNH: Hunan-Han; HDH: Huadong-Han; BJH: Beijing-Han; ZJH: Zhejiang-Han; IMR: Inner-Mongolia-Russian; SDH: Shandong-Han; LNH: Liaoning-Han; HNH-2: Henan-Han-2; GSYG: Gansu-Yugu; XJXO: Xinjiang-Xibe; HNH-1: Henan-Han-1; SCH: Sichuan-Han; SCT: Sichuan Tibetan

Supplementary Table S8. (Continue) The Fst and corresponding p value between the Sichuan Yi and 27 Chinese reference populations

Loci	Fst and p	HNH	HDH	BJH	ZJH	IMR	SDH	LNH	HNH-2	GSYG	XJXO	HNH-1	SCH	SCT
D1GATA113	Fst	-0.0021	0.0021	-0.0012	-0.0032	-0.0042	-0.0027	-0.0016	-0.0021	-0.0001	-0.0019	-0.0048	-0.0051	-0.0024
	p	0.5212	0.2683	0.4382	0.6465	0.6044	0.6003	0.4584	0.5564	0.3617	0.5188	0.7474	0.8308	0.5264
D1S1627	Fst	0.0069	0.0131	0.0050	0.0058	0.0015	0.0067	0.0019	0.0061	0.0200	0.0059	0.0131	0.0038	0.0569
	p	0.1047	0.0497	0.1440	0.1281	0.2964	0.0978	0.2686	0.1138	0.0295	0.1168	0.0603	0.1874	0.0009
D1S1677	Fst	-0.0032	-0.0031	0.0000	-0.0019	-0.0047	-0.0020	-0.0007	-0.0029	0.0145	-0.0020	-0.0047	0.0000	-0.0057
	p	0.6608	0.5968	0.3573	0.5210	0.6721	0.5357	0.4076	0.6521	0.0353	0.5561	0.7621	0.3580	0.8890
D2S441	Fst	0.0036	0.0064	0.0006	-0.0007	0.0097	0.0001	-0.0005	0.0013	0.0089	0.0031	0.0101	-0.0012	0.0008
	p	0.1657	0.1040	0.3347	0.4591	0.0949	0.3699	0.4276	0.2886	0.0820	0.1785	0.0602	0.5111	0.3459
D2S1776	Fst	0.0065	0.0084	0.0056	0.0040	-0.0026	0.0061	0.0005	0.0059	0.0086	0.0048	0.0026	0.0021	0.0056
	p	0.0875	0.0780	0.1073	0.1549	0.5618	0.0832	0.3561	0.0871	0.0817	0.1222	0.2362	0.2496	0.1261
D3S4529	Fst	-0.0017	0.0079	0.0042	-0.0012	0.0026	0.0073	0.0059	0.0089	0.0307	0.0059	0.0042	-0.0036	-0.0023
	p	0.5469	0.0952	0.1627	0.4918	0.2822	0.0660	0.1359	0.0463	0.0026	0.1047	0.1840	0.7272	0.5669
D4S2408	Fst	-0.0043	-0.0050	-0.0037	-0.0014	-0.0049	0.0001	0.0005	-0.0004	0.0001	0.0006	0.0048	-0.0050	0.0066
	p	0.8250	0.8246	0.7555	0.5024	0.6919	0.3839	0.3520	0.4184	0.3805	0.3375	0.1726	0.8879	0.1167
D5S2500	Fst	-0.0053	-0.0051	-0.0037	-0.0060	-0.0008	-0.0045	-0.0053	-0.0029	-0.0034	-0.0023	-0.0020	-0.0046	-0.0021
	p	0.9424	0.8114	0.7231	0.9962	0.4189	0.8952	0.8147	0.6589	0.6193	0.6078	0.5306	0.8274	0.5421
D6S474	Fst	0.0163	0.0035	0.0051	0.0014	-0.0069	0.0044	-0.0010	0.0065	0.0222	0.0060	-0.0027	0.0092	-0.0030
	p	0.0152	0.2070	0.1294	0.2827	0.8441	0.1471	0.4447	0.0897	0.0203	0.1022	0.5800	0.0700	0.6015
D6S1017	Fst	0.0025	-0.0034	-0.0030	0.0013	-0.0017	-0.0034	-0.0059	-0.0023	-0.0042	-0.0020	-0.0049	-0.0018	-0.0055
	p	0.2317	0.6421	0.6671	0.3067	0.4671	0.7565	0.8771	0.6122	0.7117	0.5550	0.7911	0.5389	0.8938
D9S1122	Fst	-0.0031	-0.0045	-0.0010	-0.0027	-0.0025	-0.0019	-0.0047	-0.0010	-0.0035	0.0007	0.0013	-0.0029	-0.0058
	p	0.6899	0.7620	0.4615	0.6343	0.5155	0.5655	0.7784	0.4733	0.6193	0.3280	0.3020	0.6289	0.9253
D10S1248	Fst	0.0377	0.0338	0.0422	0.0402	0.0334	0.0412	0.0360	0.0389	0.0273	0.0400	0.0319	0.0362	0.0335
	p	0.0000	0.0004	0.0000	0.0001	0.0014	0.0000	0.0002	0.0000	0.0009	0.0000	0.0005	0.0002	0.0004
D10S1435	Fst	-0.0033	-0.0057	-0.0031	0.0155	-0.0033	-0.0040	-0.0046	-0.0016	-0.0018	-0.0028	0.0024	-0.0054	-0.0057
	p	0.7569	0.9363	0.7227	0.0072	0.5989	0.8873	0.8081	0.5645	0.4890	0.7118	0.2497	0.9501	0.9197
D11S4463	Fst	-0.0018	-0.0006	-0.0029	-0.0033	-0.0056	-0.0021	-0.0036	-0.0023	0.0069	-0.0001	-0.0034	-0.0024	0.0026

	p	0.5560	0.4289	0.6933	0.7395	0.7962	0.6307	0.7221	0.6486	0.1191	0.3905	0.6954	0.6176	0.2352
D12ATA63	Fst	-0.0024	-0.0050	-0.0048	-0.0017	-0.0043	-0.0031	-0.0017	-0.0018	0.0375	-0.0029	0.0007	-0.0046	0.0056
	p	0.6228	0.8467	0.9082	0.5406	0.6736	0.7249	0.4994	0.5780	0.0004	0.6983	0.3322	0.8584	0.1374
D14S1434	Fst	-0.0002	-0.0042	0.0045	-0.0015	0.0072	0.0000	-0.0007	0.0009	-0.0022	-0.0005	0.0024	-0.0008	-0.0059
	p	0.3877	0.7393	0.1476	0.5022	0.1425	0.3862	0.4204	0.3074	0.5266	0.4212	0.2531	0.4329	0.9269
D17S1301	Fst	0.0014	0.0027	-0.0004	-0.0014	-0.0028	0.0014	-0.0027	0.0004	0.0127	-0.0008	-0.0016	0.0006	0.0100
	p	0.2752	0.2346	0.4068	0.4959	0.5503	0.2707	0.6103	0.3446	0.0523	0.4452	0.5206	0.3330	0.0676
D18S853	Fst	-0.0047	-0.0034	-0.0037	-0.0022	-0.0079	-0.0021	-0.0030	-0.0029	-0.0017	-0.0029	0.0065	-0.0033	-0.0009
	p	0.9046	0.6593	0.7633	0.5828	0.9491	0.5955	0.6128	0.7008	0.4909	0.7066	0.1215	0.6989	0.4378
D19S433	Fst	-0.0023	-0.0009	-0.0022	-0.0026	-0.0071	-0.0010	-0.0021	-0.0020	0.0095	-0.0033	-0.0023	-0.0036	-0.0009
	p	0.6870	0.4874	0.6538	0.7276	0.9658	0.5143	0.5982	0.6580	0.0477	0.8515	0.6316	0.8193	0.4954
D20S482	Fst	0.0008	0.0002	0.0035	0.0038	0.0004	-0.0006	0.0136	0.0010	0.0126	-0.0007	-0.0013	0.0103	-0.0053
	p	0.3237	0.3741	0.1894	0.1798	0.3562	0.4410	0.0342	0.3060	0.0499	0.4415	0.4769	0.0479	0.8704
D22S1045	Fst	0.0177	0.0149	0.0067	0.2997	0.0822	0.0085	0.0004	0.0074	0.0021	0.0043	0.0050	0.0143	-0.0039
	p	0.0070	0.0192	0.0908	0.0000	0.0000	0.0513	0.3615	0.0641	0.2656	0.1480	0.1709	0.0183	0.7187

LST: Lhasa-Tibetan; QHS: Qinghai-Salar; HBTJ: Hubei-Tujia; YNB: Yunnan-Bai; NXH: Ningxia-Han; AU: Kuqa-Uyghur; ILK: Ili-Kazakh; FJS: Fujian-She; IMM: Inner-Mongolia-Mongolian; NH: Northern-Han; GNH: Guanzhong-Han; YNY: Yunnan-Yi; HNL: Hainan-Li; GGH: Guangdong-Han; HNH: Hunan-Han; HDH: Huadong-Han; BJH: Beijing-Han; ZJH: Zhejiang-Han; IMR: Inner-Mongolia-Russian; SDH: Shandong-Han; LNH: Liaoning-Han; HNH-2: Henan-Han-2; GSYG: Gansu-Yugu; XJXO: Xinjiang-Xibe; HNH-1: Henan-Han-1; SCH: Sichuan-Han; SCT: Sichuan Tibetan

Supplementary Table S9. The pairwise Nei's genetic distance among 28 Chinese populations

Populations	[01]	[02]	[03]	[04]	[05]	[06]	[07]	[08]	[09]	[10]	[11]	[12]	[13]	[14]	[15]	[16]	[17]	[18]	[19]	[20]	[21]	[22]	[23]	[24]	[25]	[26]	[27]	[28]
[01] SCY																												
[02] SCT	0.0282																											
[03] XJXB	0.0280	0.0276																										
[04] HNH-1	0.0450	0.0433	0.0343																									
[05] SCH	0.0221	0.0253	0.0143	0.0290																								
[06] LST	0.0299	0.0209	0.0309	0.0478	0.0260																							
[07] QHS	0.0347	0.0331	0.0286	0.0454	0.0228	0.0355																						
[08] HBTJ	0.0281	0.0285	0.0236	0.0375	0.0094	0.0264	0.0340																					
[09] YNB	0.0280	0.0274	0.0185	0.0385	0.0121	0.0268	0.0307	0.0159																				
[10] NXH	0.0242	0.0247	0.0165	0.0295	0.0074	0.0239	0.0277	0.0131	0.0120																			
[11] AU	0.0245	0.0306	0.0193	0.0382	0.0083	0.0283	0.0283	0.0158	0.0167	0.0131																		
[12] ILK	0.0391	0.0306	0.0174	0.0470	0.0274	0.0281	0.0382	0.0273	0.0228	0.0257	0.0214																	
[13] FJS	0.0552	0.0577	0.0545	0.0688	0.0423	0.0544	0.0331	0.0469	0.0437	0.0476	0.0523	0.0645																
[14] IMM	0.0407	0.0398	0.0233	0.0481	0.0247	0.0406	0.0372	0.0332	0.0310	0.0281	0.0297	0.0372	0.0651															
[15] NH	0.0321	0.0313	0.0184	0.0385	0.0112	0.0340	0.0158	0.0172	0.0178	0.0152	0.0193	0.0313	0.0365	0.0268														
[16] GNH	0.0248	0.0235	0.0118	0.0296	0.0064	0.0224	0.0232	0.0115	0.0120	0.0054	0.0129	0.0232	0.0467	0.0218	0.0114													
[17] YNY	0.0494	0.0553	0.0399	0.0657	0.0326	0.0542	0.0310	0.0400	0.0408	0.0384	0.0356	0.0462	0.0400	0.0520	0.0314	0.0389												
[18] HNL	0.0424	0.0534	0.0439	0.0560	0.0173	0.0533	0.0464	0.0274	0.0336	0.0270	0.0176	0.0582	0.0582	0.0486	0.0336	0.0279	0.0500											
[19] GGH	0.0267	0.0356	0.0225	0.0392	0.0056	0.0360	0.0312	0.0111	0.0187	0.0123	0.0092	0.0374	0.0465	0.0332	0.0184	0.0131	0.0348	0.0120										
[20] HNH	0.0246	0.0287	0.0174	0.0321	0.0034	0.0305	0.0252	0.0091	0.0148	0.0097	0.0098	0.0314	0.0410	0.0275	0.0123	0.0077	0.0338	0.0152	0.0041									
[21] HDH	0.0264	0.0290	0.0159	0.0334	0.0059	0.0294	0.0218	0.0119	0.0135	0.0085	0.0131	0.0303	0.0370	0.0253	0.0096	0.0082	0.0273	0.0231	0.0080	0.0053								
[22] BJH	0.0233	0.0241	0.0123	0.0279	0.0044	0.0233	0.0226	0.0104	0.0110	0.0067	0.0119	0.0249	0.0443	0.0235	0.0093	0.0047	0.0354	0.0237	0.0108	0.0053	0.0056							
[23] ZJH	0.0241	0.0249	0.0156	0.0299	0.0065	0.0299	0.0255	0.0120	0.0158	0.0106	0.0135	0.0272	0.0448	0.0261	0.0131	0.0081	0.0346	0.0252	0.0112	0.0079	0.0092	0.0061						
[24] IMR	0.0395	0.0434	0.0279	0.0482	0.0237	0.0478	0.0185	0.0374	0.0304	0.0251	0.0290	0.0449	0.0409	0.0388	0.0133	0.0239	0.0282	0.0462	0.0318	0.0262	0.0184	0.0215	0.0272					
[25] SDH	0.0216	0.0208	0.0108	0.0274	0.0041	0.0229	0.0223	0.0085	0.0108	0.0056	0.0115	0.0231	0.0417	0.0221	0.0095	0.0035	0.0360	0.0250	0.0094	0.0044	0.0046	0.0018	0.0052	0.0234				
[26] LNH	0.0233	0.0219	0.0118	0.0296	0.0064	0.0239	0.0237	0.0134	0.0113	0.0077	0.0148	0.0234	0.0441	0.0254	0.0124	0.0064	0.0393	0.0284	0.0134	0.0095	0.0095	0.0049	0.0092	0.0266	0.0046			
[27] HNH-2	0.0221	0.0215	0.0102	0.0272	0.0042	0.0226	0.0227	0.0096	0.0116	0.0052	0.0113	0.0222	0.0427	0.0228	0.0091	0.0036	0.0342	0.0252	0.0098	0.0043	0.0048	0.0018	0.0063	0.0219	0.0013	0.0044		
[28] GSYG	0.0454	0.0381	0.0333	0.0469	0.0320	0.0322	0.0427	0.0334	0.0353	0.0268	0.0361	0.0352	0.0661	0.0472	0.0399	0.0278	0.0630	0.0569	0.0355	0.0347	0.0293	0.0299	0.0342	0.0503	0.0265	0.0311	0.0272	

LST: Lhasa-Tibetan; QHS: Qinghai-Salar; HBTJ: Hubei-Tujia; YNB: Yunnan-Bai; NXH: Ningxia-Han; AU: Kuqa-Uyghur; ILK: Ili-Kazakh; FJS: Fujian-She; IMM: Inner-Mongolia-Mongolian;

NH: Northern-Han; GNH: Guanzhong-Han; YNY: Yunnan-Yi; HNL: Hainan-Li; GGH: Guangdong-Han; HNH: Hunan-Han; HDH: Huadong-Han; BJH: Beijing-Han; ZJH: Zhejiang-Han; IMR: Inner-Mongolia-Russian; SDH: Shandong-Han; LNH: Liaoning-Han; HNH-2: Henan-Han-2; GSYG: Gansu-Yugu; XJXO: Xinjiang-Xibe; HNH-1: Henan-Han-1; SCH: Sichuan-Han; SCT: Sichuan Tibetan; SCY: SiChuan Yi
